# The neuroimmune microenvironment of peripheral nerve injury: mechanisms, pathophysiology, and therapeutic implications

**DOI:** 10.3389/fsurg.2026.1745990

**Published:** 2026-02-16

**Authors:** Wesley S. Warner, Madeline Rose, Stewart Yeoh, Whitney E. Muhlestein, Sama Noroozi Gilandehi, Mark A. Mahan

**Affiliations:** 1 Interdepartmental Program in Neuroscience, University of Utah, Salt Lake City, UT, United States; 2 Department of Neurosurgery, Clinical Neurosciences Center, University of Utah, Salt Lake City, UT, United States

**Keywords:** neuroimmune, neuroinflammation, neuroma, peripheral nerve injury, peripheral nervous system, regeneration, translational therapies, Wallerian degeneration

## Abstract

The peripheral nervous system has the remarkable capacity for spontaneous regeneration after injury. Despite this inherent capability, clinical outcomes remain poor and are often hallmarked by pathophysiologic neuroma formation and limited neurologic recovery. Inflammation is fundamental for successful regeneration but can propagate pathophysiologic outcomes when aberrantly activated. Although the numerous mechanisms whereby nerve regeneration is derailed into a pathophysiologic state have yet to be established, a growing body of research has elaborated the interplay of neuroimmune interactions in successful nerve regeneration. In this review, we synthesize the current understanding of neuroimmune interactions in traumatic peripheral nerve injury, regeneration, and pathophysiology across three domains: (1) resident immune response; (2) innate immune response; and (3) adaptive immune response. Here, we examine the temporal dynamics of immune cell recruitment, polarization, and functional contributions during Wallerian degeneration and regeneration. We propose potential mechanisms of pathophysiologic regeneration, including failed inflammatory resolution and neuroimmune interactions that sustain maladaptive responses. Finally, we aim to connect these basic science mechanisms to current therapeutic strategies. Specifically, we detail how pharmacologic interventions, cellular therapies, energetic stimulation, and hydrogel or conduir-based approaches may modulate the immune response and shape the microenvironment to improve regenerative outcomes. Collectively, a comprehensive understanding of the bidirectional interactions among neural, immune, and other local cell types within the injury microenvironment is critical for developing strategies to improve nerve regeneration and neurologic outcomes.

## 1 Introduction

**Figure 1:**
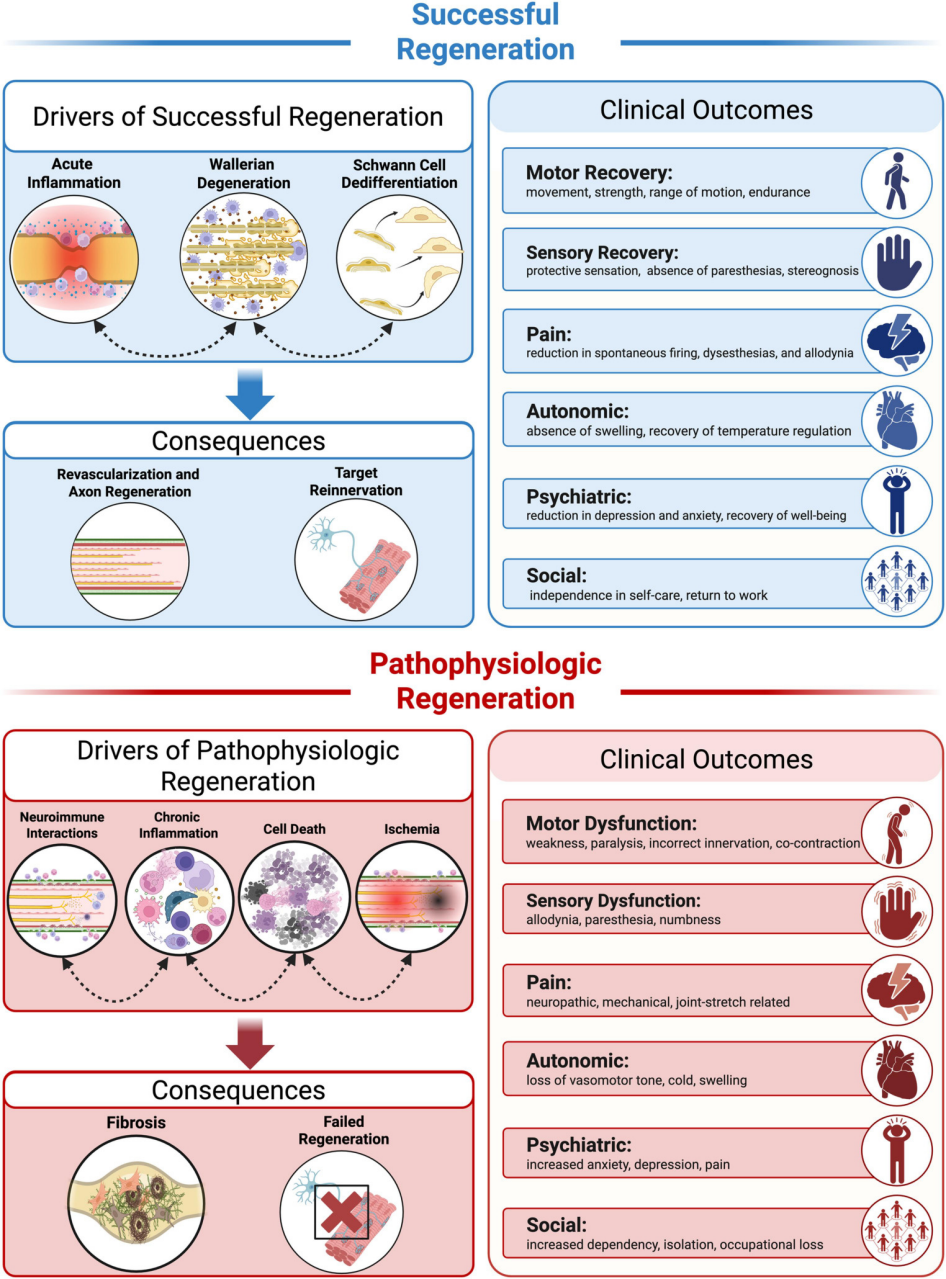
Physiologic drivers, consequences, and clinical outcomes of successful vs. pathophysiologic nerve regeneration. Successful regeneration (top): Coordinated biological processes including acute inflammation, Wallerian degeneration, and Schwann cell dedifferentiation facilitate revascularization, axon regeneration, and target reinnervation. These processes are associated with positive clinical outcomes across motor, sensory, autonomic, psychiatric, and social domains. Pathophysiologic regeneration (bottom): Interconnected pathologic processes including aberrant neuroimmune interactions, chronic inflammation, cell death, and ischemia result in fibrosis and failed regeneration. Associated clinical outcomes include motor and sensory dysfunction, neuropathic pain, and impaired quality of life. Figure created with BioRender.com.

Peripheral nerve injuries can result in profound neurologic disability and are frequently associated with permanent functional and sensory deficits (1, 2). These deficits are often coupled with the development of comorbidities such as chronic pain, anxiety, and depression (Figure 1) (3). Approximately 2%–3% of trauma patients sustain a severe nerve injury, with over 18,000 major nerve injuries occurring annually in the United States—an incidence exceeding that of spinal cord injury (4, 5). Severe peripheral nerve injury mechanisms include laceration, crush, and compression; however, nearly 80% arise from high-speed trauma that produces abrupt acceleration–deceleration forces on the nerve (6–8). Currently, the only treatment option to convert non-regenerative injuries into regenerative outcomes is through surgical intervention. Unfortunately, despite advances in surgical techniques and postoperative care, outcomes for severe peripheral nerve injuries are often unsatisfactory (9, 10). Furthermore, although many drugs, devices, and therapies have demonstrated promise in the laboratory setting, few treatments to improve nerve regeneration are available for clinical use.

Unlike the central nervous system (CNS), the peripheral nervous system (PNS) has remarkable regenerative capacity (11, 12); however, in human injuries this regeneration is often incomplete, and a common sequela is the pathophysiologic development of a neuroma or neuroma-in-continuity (Figure 1) (13, 14). This pathology is a form of frustrated regeneration, whereby axons attempt to regrow but become encapsulated in fibrotic tissue (15–17). Axons are unable to navigate this inhibitory microenvironment and fail to reach their distal targets, often resulting in persistent neurologic deficits (14, 18). Furthermore, this pathophysiology is the predominant clinical outcome, but it has not been recapitulated in a basic science model until recently (19–22). Therefore, we have a limited understanding of the cellular and molecular underpinnings orchestrating pathophysiologic nerve regeneration.

Neuroinflammation is a critical component of successful regeneration whereby immune cells are recruited to the injury site to clear cellular and myelin debris in a process known as Wallerian degeneration (WD) (23–25). In addition to debris clearance, immune cells also contribute to revascularization and axonal regrowth through the secretion of cytokines and trophic factors after injury (26–28); however, the immune response can also become maladaptive and aberrantly activated, as observed in contexts such as neurodegeneration and cancer (29, 30). Both human neuroma specimens and experimental models reveal persistent hypercellularity, sustained immune cell infiltration, and robust fibrotic deposition within the neuroma environment (16, 31–33). Thus, dysregulated inflammation may serve as an inciting event in driving pathophysiologic outcomes. Despite expanded insight of the signaling cascades underpinning favorable regeneration, the temporal and spatial coordination of immune responses remain incompletely understood (34–36). Moreover, there is limited interrogation into the inflammatory programs governing pathophysiologic nerve regeneration. Therefore, elucidating these mechanisms may uncover novel therapeutic targets to prevent maladaptive outcomes.

In this review, we examine the basic science literature characterizing the inflammatory response to peripheral nerve injury. First, we provide a primer on the architecture of peripheral nerves. Next, we delineate the resident immune cell landscape and the immediate inflammatory cascade within the nerve microenvironment. Then, we describe the sequential activation of innate and adaptive immune responses, emphasizing key mechanistic insights and unresolved questions. Finally, given the absence of clinically approved therapies that augment nerve regeneration, we summarize current preclinical therapeutics that leverage immunomodulatory strategies to improve successful regeneration. Collectively, these insights may inform future translational strategies aimed at guiding the immune response toward successful nerve regeneration.

## 2 Peripheral nerve anatomy

### 2.1 Structural organization

Peripheral nerves are intricate structures composed of multiple layers of cellular elements that facilitate efficient signal transmission while simultaneously providing both mechanical and metabolic support (37). The primary units within the PNS are the axons, which transmit electrical impulses between the CNS and their peripheral targets. Large-diameter axons (typically >1.0 μm) are myelinated, whereas smaller-diameter axons are typically unmyelinated and are associated with nonmyelinating Schwann cells (SCs) recognized as Remak bundles (38). Axons are organized within a multilayered connective tissue framework that provides both structural support and a robust microenvironment for metabolic exchange. The endoneurium is the innermost connective tissue layer, surrounding both individual axons and their associated SCs. The perineurium encases the endoneurium to form fascicles, creating a protective sheath that contributes to the blood–nerve barrier (BNB). Finally, the epineurium is the outermost connective tissue layer that surrounds the entire nerve, both providing structural support and functioning as a shield to help protect the nerve from mechanical stress and injury (39).

### 2.2 Blood–nerve barrier

The BNB is a specialized diffusion barrier that maintains the ionic homeostasis of peripheral nerves by tightly regulating leukocyte and solute trafficking. Structurally, the BNB consists of two complementary components: the perineural barrier and the epineurial microvascular barrier. The perineural barrier is formed by multiple concentric layers of perineurial glia interconnected by tight junction-rich proteins such as claudin-1, occludin, and zonula occludens-1 (40). Furthermore, these cells express selective transporters such as glucose transporter type 1 and active transcytotic machinery that helps regulate endoneurial access of leukocytes and plasma proteins (41). The epineural microvascular barrier is composed of non-fenestrated endothelial cells that are supported by pericytes and a basement membrane (42). Under typical conditions, these barriers tightly regulate the movement of proteins, plasma, ions, and immune cells into the endoneurial compartment, therefore preserving a metabolically and electrically stable microenvironment that is essential for axonal conduction and SC function.

## 3 Resident immune response

### 3.1 Inflammatory signaling

**Figure 2:**
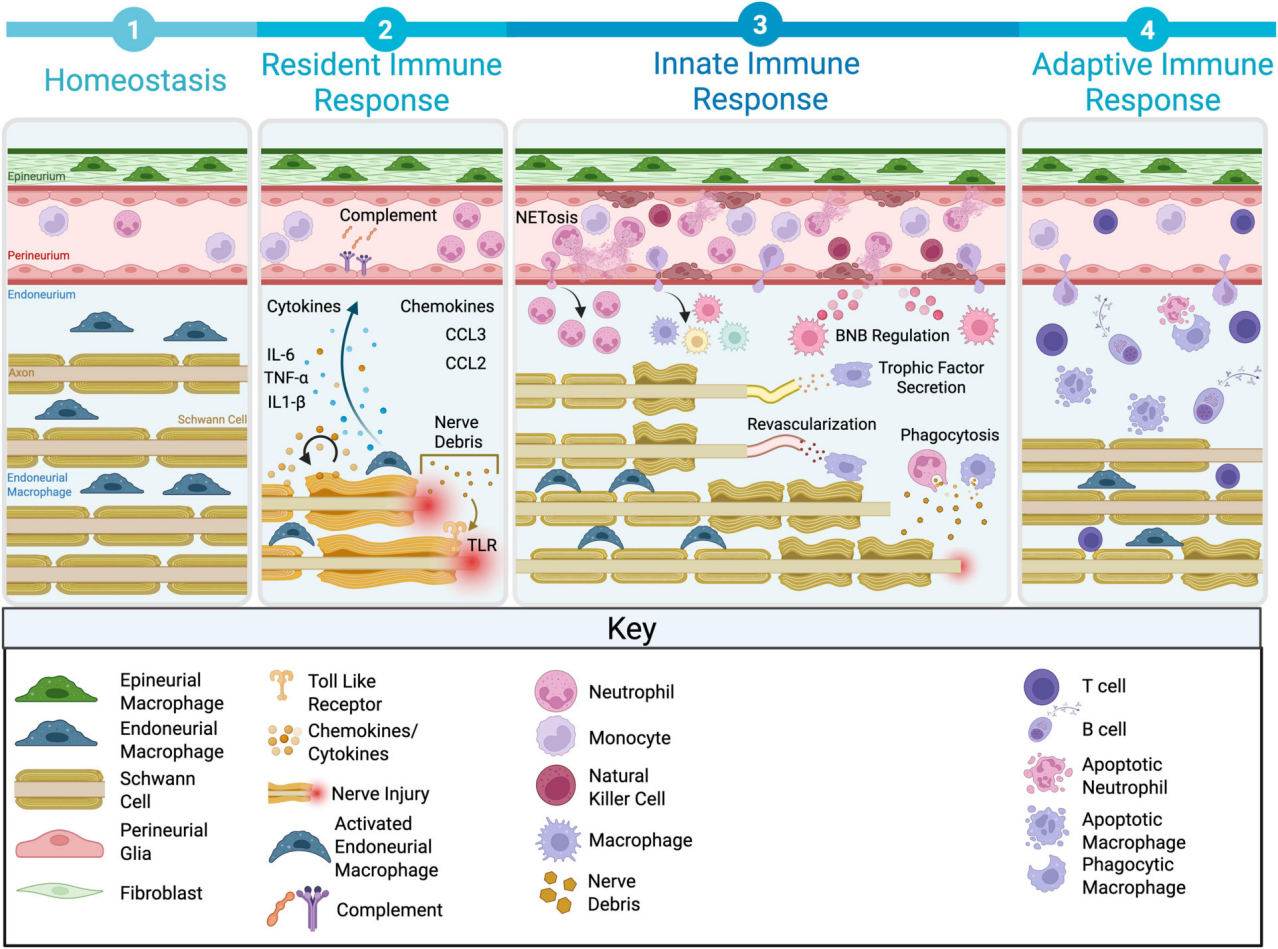
Neuroinflammatory response to peripheral nerve injury. (1) Homeostasis depicts the baseline state of the uninjured nerve with resident immune and glial cells. (2) The resident immune response involves early complement activation, cytokine release (IL-6, TNF-α, IL-1β), and chemokine signaling (CCL3, CCL2). (3) The innate immune response is characterized by neutrophil infiltration and NETosis, phagocytosis of nerve debris, blood–nerve barrier modulation, trophic factor secretion, and revascularization to support regeneration. (4) The adaptive immune response involves T cell and B cell recruitment, contributing to tissue remodeling and resolution of inflammation. IL, interleukin; TNF, tumor necrosis factor; CCL, monocyte chemoattractant protein-1 (MCP-1); TLR, toll-like receptor; BNB, blood–nerve barrier. Figure created with BioRender.com.

The earliest chemokines and cytokines expressed during WD are proinflammatory. Tumor necrosis factor-α (TNF-α), whose messenger RNA (mRNA) and protein are expressed constitutively at low levels in the intact PNS, is rapidly upregulated in SCs after nerve injury (Figure 2) (43). This is followed by increased expression of interleukin (IL)-6 from local fibroblasts and then by upregulation of IL-1α, IL-1β, and IL-6 in SCs. About 24 h after peripheral nerve injury, there is a rapid increase in the expression of mRNA for the potent macrophage chemokines monocyte chemoattractant protein-1 (MCP-1/CCL2) and macrophage inflammatory protein-1α (MIP-1α/CCL3) (44). Together, MCP-1 and MIP-1α are responsible for approximately 80% of macrophage recruitment to the injured sciatic nerve in mice. The upregulation of both of these chemokines may be induced by TNF-α and/or IL-1β, but there is particularly strong evidence that IL-6 produced by SCs acts in an autocrine fashion to increase expression of MCP-1 and macrophage recruitment (45). Infiltrating macrophages are then induced by SC-derived cytokines to produce their own TNF-α, IL-1α, IL-1β, and IL-6, creating a positive feedback loop that continues to attract macrophages to the site of nerve injury.

This inflammatory cascade is initiated by innate immune recognition of axonal and myelin debris products. After nerve injury, there is an accumulation of the byproducts of calpain-mediated axonal degeneration at the site of injury, including dying cells, heat shock proteins, and components of the extracellular matrix (ECM) (Figure 2). These liberated ligands may act on toll-like receptors (TLRs) expressed by SCs, activating downstream signaling pathways to induce expression of chemokines and cytokines (46). Boivin and colleagues demonstrated that mice deficient in TLR2, TLR4, and their downstream signaling protein MYD88 exhibit diminished expression of IL-1β and MCP-1 after sciatic nerve injury (47).

In collaboration with cytokine-mediated macrophage recruitment, the complement system is activated rapidly (within an hour) after injury to promote debris opsonization and clearance (Figure 2). Complement component C1q binds components of degraded myelin and axons, which are then opsonized by C4b, C3b, and C5b, targeting the debris for phagocytosis (48). Depletion of C3 blocks myelin phagocytosis and delays WD (49, 50), whereas deficiency of C5 and C6, which are critical for the formation of the membrane attack complex, is associated with decreased macrophage recruitment and myelin phagocytosis (48, 51).

As WD progresses, this proinflammatory environment is accompanied by increased expression of anti-inflammatory cytokines, particularly transforming growth factor (TGF)-β and IL-10. TGF-β is expressed by SCs and induces their proliferation while also blocking their transition to a myelinating phenotype (52). IL-10 is secreted at low, ineffective levels by resident fibroblasts within 5 h of injury. However, as recruited macrophages enter the site of injury they express higher levels of IL-10, with protein expression peaking around day 7 (53). Negative feedback from IL-10 limits the secretion of cytokines including TNF-α and IL-1α/-1β, which then limits the proliferation of macrophages.

### 3.2 Blood-nerve barrier and immune cell entry

The BNB is a putative inhibitor to infiltrating immune cells. Adhesion molecules facilitate the transmigration of hematogenous cells into inflamed tissues. Intracellular adhesion molecule-1 (ICAM-1) binds endothelial cells and acts as a ligand for complement receptor 3, macrophage-1 antigen, and leukocyte function-associated molecule on macrophages. After nerve injury, cytokines, including TNF-α, the IL-1 family, and IL-6, are thought to upregulate ICAM-1, potentially increasing the permeability of injured nerves to circulating immune cells. Thus, mice deficient in ICAM-1 expectedly exhibit diminished recruitment of macrophages, but interestingly, these mice partially compensate for fewer recruited macrophages with greater proliferation of SCs and resident macrophages (54). Endothelial activation via nuclear factor-κB signaling and pericyte detachment further destabilize the microvascular barrier, thereby amplifying local inflammation. Importantly, in addition to allowing immune infiltration, BNB dysfunction also sustains a feed-forward loop of cytokine signaling that hinders remyelination and axonal regeneration. This suggests that vascular permeability may be linked to the persistence of neuroinflammatory states (55). Therefore, the BNB serves as both an inhibitor and amplifier of peripheral neuroinflammation.

### 3.3 Resident cell contributions

#### 3.3.1 Schwann cells

SCs play a central role in the early stages of WD by initiating repair responses that support regeneration. Myelinating SCs, which typically form the insulating myelin sheath, rapidly convert to a repair phenotype after nerve injury. This repair phenotype involves downregulation of myelin-associated genes, upregulation of regeneration-associated transcription factors, secretion of neurotrophic factors and chemokines, expression of innate immune receptors, and participation in myelin clearance and antigen presentation (56, 57). Because of early secretion of cytokines and chemokines, SCs recruit and activate macrophages, in turn triggering the cascade of degeneration-associated inflammation that is also essential for subsequent regeneration (58). Non-myelinating (Remak) SCs ensheathe small-diameter axons and exhibit distinct transcriptional programs compared with myelinating SCs. Remak SCs provide metabolic support to nociceptive and autonomic fibers, upregulate immune-related genes, and importantly can adopt repair-like states after injury that promote chemokine production and cross-talk with macrophages (59). Collectively, SCs orchestrate several important functions during the immediate response to nerve injury and subsequent regeneration. Although not the primary focus of this review, these functions have been discussed in greater detail elsewhere (60–63).

#### 3.3.2 Perineurial glia

Although perineurial glia have been less extensively studied than SCs or macrophages, live-imaging studies in zebrafish models have demonstrated that they contribute to the resident cellular response after nerve transection injury (38, 64, 65). Perineurial glia facilitate two cardinal functions after nerve injury: (1) phagocytosis of debris and (2) formation of cellular bridges across injury gaps. Lewis and Kucenas observed that perineurial glia rapidly extend processes toward damaged sites and phagocytose debris immediately after laser-induced nerve transection injury (64). In addition, time-lapse imaging revealed that perineurial glia sequentially localize to the proximal and then distal degenerating stumps around the injury gap, whereas macrophages appeared to home to the injury zone specifically. The second finding identified that perineurial glia traverse the injury gap to form scaffold-like structures in advance of SCs and axonal regeneration (64). Notably, when the size of the gap is larger, perineurial bridge formation fails, and correspondingly, a lack of axonal regeneration is observed. In subsequent experiments, genetic inhibition of perineurial glia development resulted in an absence of bridge formation, with nearly half of nerves failing to regenerate axons after injury. Further mechanistic perturbations demonstrated that this bridging phenomenon is mediated by TGF-β signaling, with SCs acting synergistically with perineurial glia to facilitate bridge formation (65). A role for perineurial glia that is similar to regeneration has also been observed in a rodent models; after a nerve gap injury, perineurial glia migrate into conduits by 7 days after injury, followed by blood vessels, SCs, and axons in a collaborative effort to rebuild the nerve microenvironment (66). However, because these observations were based primarily on electron microscopy studies, further molecular characterization of perineurial glia is needed to confirm their identity and function.

## 4 Innate immune response

### 4.1 Neutrophils

Neutrophils are the first innate immune cells to infiltrate the lesion after peripheral nerve injury (67, 68), appearing within hours and peaking in number 1–3 days after-injury (Figure 2). Early investigation focused on their potential role in contributing to nerve injury-induced hyperalgesia and demonstrated that depleting neutrophils around the time of their normal infiltration resulted in attenuated heat-induced hyperalgesia (67).

To further define the role of neutrophils in WD and regeneration, investigators examined CCR2^−/−^ mice with impaired monocyte recruitment. Because monocytes give rise to recruited macrophages and encompass the largest macrophage population during WD, it was hypothesized that neutrophils might compensate for macrophage function in this genotype (69–71). Characterization of WD in CCR2^−/−^ mice demonstrated that myelin clearance was unimpaired and revealed that this process was mediated by neutrophils, which were increased in number, sustained in the nerve, and accompanied by persistent expression of neutrophil-associated chemokines (72). Furthermore, using an antibody to deplete neutrophils, myelin removal was reduced in both wild-type and CCR2^−/−^ animals, with additive effects observed in the CCR2^−/−^ genotype (72). These experiments revealed that neutrophils play a critical role in phagocytosis of myelin after nerve injury and likely collaborate synergistically with macrophages to coordinate debris clearance over the course of WD.

Neutrophils not only act as professional phagocytes but also exert effector functions such as granule release and NETosis, warranting further investigation of their roles in peripheral nerve injury (73). NETosis is a form of cell death hallmarked by the expulsion of decondensed chromatin and granules to entrap pathogens (74). Although NETosis serves a protective role in infection, aberrant neutrophil extracellular trap (NET) formation in neurologic injury contexts such as traumatic brain injury and stroke results in increased fibrosis and poor neurologic outcomes, which can be rescued by inhibiting NET formation (75–77). This suggests a detrimental role of NETs in pathophysiologic progression of neurologic injury. Despite these novel findings in CNS injury, the role of NETosis in peripheral nerve trauma remained unexplored until recently. Yamamoto et al. identified that NETs formed on the exterior of the nerve after crush injury (76). Blocking NET formation or degrading NETs enhanced macrophage infiltration into the nerve, suggesting that NETs act as a barrier to macrophage entry. Inhibition of migration inhibitory factor, which can induce NET formation, was associated with decreased myelin debris and improved axon regeneration at 7 days post injury (DPI) (76). Interestingly, these results contrast with those from another study reporting that NET inhibition attenuated myelin clearance at 7 DPI relative to vehicle controls (78). Discrepancies between studies likely stem from several methodological differences including injury models, NET inhibitors, and delivery routes, which may account for the conflicting outcomes regarding myelin clearance and regeneration. Furthermore, neither study comprehensively assessed functional neurologic recovery (sensory and motor) in the absence of NET formation.

The role of neutrophils in peripheral nerve regeneration warrants further investigation. Because they are the first responders to injury, the interaction of neutrophils within the injury microenvironment subsequently impacts cross-talk with other cells and likely plays an important role in orchestrating the immediate inflammatory response to injury (79). In the context of severe peripheral nerve injury, neutrophil response via the secretion of NETs and subsequent exacerbation of inflammation potentially detracts from their role as phagocytes and synergistic collaboration with macrophages. Furthermore, a cardinal process mediating inflammatory resolution and promoting an anti-inflammatory milieu is efferocytosis, in which macrophages engulf and phagocytose apoptotic leukocytes (80). If neutrophils undergo NETosis rather than programmed cell death, efferocytosis may be impeded, thereby contributing to failed inflammatory resolution. Beyond NET formation, important questions remain regarding how neutrophils may interact with SCs, axons, and nerve vasculature at the injury site and over the course of nerve regeneration.

### 4.2 Macrophages

Macrophages serve as immune sentinels that play critical roles during both homeostasis and disease. In other tissues, especially the well-studied CNS, resident macrophage populations perform immune surveillance, maintain tissue barrier integrity, and also provide trophic support to the local tissue environment (81). Furthermore, macrophages are remarkably plastic, responding to environmental signals by adopting diverse transcriptional profiles and functional states (82). These functions are dramatically altered in the context of peripheral nerve injury, in which macrophages contribute significantly to phagocytosis of debris, recruitment/activation of other immune cells, and secretion of trophic factors (Figure 2). Although these dynamic transitions are thought to be in part locally driven, intrinsic factors such as ontogeny and prior experience likely contribute to imprinting distinct response programs (83, 84). In this section, we elaborate on the spatial organization of resident macrophage populations in the PNS, how non-resident macrophages are recruited and activated after injury, the phenotypic and molecular diversity of macrophages, and their functional roles during acute injury and repair phases.

#### 4.2.1 Spatial organization and developmental origins of resident macrophages

To understand macrophage contributions to nerve injury and repair it is essential to first characterize the resident populations present during homeostasis. Although resident macrophages have previously been identified in the PNS (26, 85, 86), their developmental origins, homeostatic turnover, and function have remained largely unexplored.

To determine the developmental origins of PNS resident macrophages, lineage tracing studies using Cx3cr1CreERT2:Rosa26-YFP mice tagged at embryonic day 16.5 revealed that approximately 60% of sciatic nerve macrophages (snMacs) at postnatal day 4 were embryonically derived (71). However, by postnatal day 42, only half the resident macrophage population were positively labeled, suggesting a postnatal window of turnover. To test this theory, Ydens and colleagues used S100a4Cre:Rosa26-YFP mice to label hematopoietic stem cell–derived monocytes and found that snMac labeling doubled between 2 and 6 weeks postnatally, indicating that snMacs are established embryonically but are gradually replaced by hematopoietic stem cell-derived monocytes over the course of development. As in the adult CNS, resident macrophage turnover under homeostatic conditions was limited, with resident macrophage populations observed to be maintained through at least 36 weeks of age (71). Therefore, unlike CNS microglia or CNS-associated macrophages, resident PNS macrophages do not appear to be solely yolk sac-derived (83).

Expanding on studies of developmental origins and turnover dynamics, advances in single-cell technologies have revealed spatial and molecular heterogeneity within the resident macrophage populations. Notably, single-cell RNA sequencing (scRNA-seq) has permitted the identification of two distinct macrophage subsets: epineurial macrophages (Relmα^+^Mgl1^+^) and endoneurial macrophages (Relmα^−^Mgl1^−^) (71). Endoneurial macrophages share genetic signatures more closely aligned with microglia than with epineurial macrophages, suggesting that proximity to axons and SCs promotes a microglia-like phenotype. This initial distinction of two resident macrophage populations has been corroborated by additional single-cell sequencing studies (87, 88). Furthermore, these findings in the PNS parallel observations in other tissues, such as lung and brain, where local environmental niches appear to instruct macrophage identity and fate specification (89, 90).

Despite these findings, significant challenges remain in studying these resident macrophage populations. Notably, because identification currently relies on a combination of markers together with anatomical context, and because gene expression profiles and canonical markers are dynamically regulated over the course of injury and recovery, it remains challenging to definitively link specific macrophage populations to distinct injury-related functions. Furthermore, the cues that drive the fate specification of these distinct subsets, how these populations are maintained, and their functional roles during homeostasis remain poorly understood.

#### 4.2.2 Monocyte-derived macrophages: recruitment and differentiation after injury

Early studies in the 1980s questioned whether recruited immune cells contribute to WD. Beuche and Friede addressed this question by placing injured sciatic or phrenic nerves into Millipore diffusion chambers with varying pore sizes to restrict cellular infiltration before implanting them into the peritoneal cavity for 8 weeks (91). In chambers that prevented leukocyte entry, no increase in cell density was observed and myelin was largely maintained. By contrast, in chambers permitting leukocyte infiltration, cell increases were detected by 3 days post-transplantation, with many cells appearing “myelinophagic” by 1 week. These explanted nerves recapitulated features of WD observed *in vivo*, with marked myelin reduction by 4 weeks after insertion (91). Follow-up studies using silica injections to physically block cellular infiltration aimed to characterize the cellular identity of the infiltrating cells and test whether these cells precipitate WD (86). Blocking cell entry significantly reduced both cell density and myelin debris clearance immediately after peripheral nerve injury. Furthermore, MAC-1 (CD11b) and F4/80 staining identified the infiltrating cells as myeloid cells and macrophages, and subsequent studies demonstrated that recruited monocytes differentiate into macrophages upon entering the PNS (69, 70, 85, 92, 93).

These experiments demonstrated that recruited immune cells substantially contribute to WD and were later expanded upon with technological advances. Notably, scRNA-seq identified a substantial macrophage population at 1 day after crush injury that was absent in naïve nerve tissue, suggesting robust early recruitment after injury (71). This finding was corroborated using a fate-mapping approach in which irradiated mice (with the surgical limb shielded) were transplanted with CD45.1 bone marrow. Resident macrophages were still detectable in the nerve, yet by 1 day after injury a large proportion of CD45.1^+^ Ly6C^+^ monocytes had infiltrated the endoneurium (71). Subsequently, these cells downregulated Ly6C and adopted macrophage phenotypes. Interestingly, similar to observations in the CNS (94), there appears to be potential for monocytes to repopulate the peripheral nerve after injury and contribute to homeostatic resident macrophage population thereafter (71); however, the cues that permit monocyte imprinting into the peripheral niche remain unexplored.

Deconstructing the roles of infiltrating vs. resident macrophages remains challenging because of limited lineage-specific markers. Once recruited to the injured nerve, monocytes downregulate markers such as CCR2 and Ly6C (bone marrow–derived monocyte markers) and acquire macrophage signatures, which complicates efforts to distinguish resident from peripherally derived macrophages during the distinct phases of nerve regeneration (71). In addition, studies that broadly deplete macrophages to assess impact on WD may present confounding results because of simultaneous impact on resident, recruited, and CNS macrophage populations. Although research efforts have elaborated important insights into the contributions of recruited macrophages to injury and repair, the distinct contributions of specific subtypes (e.g., recruited vs. resident) are likely more nuanced than is currently appreciated.

#### 4.2.3 Macrophage polarization profiles: phenotypic and molecular diversity

Macrophages in the peripheral nerve demonstrate robust phenotypic diversity across the course of nerve injury and regeneration. Macrophages have historically been categorized as predominantly M1 (pro-inflammatory) and M2 (anti-inflammatory); however, with improved sequencing technologies, it is clear that this binary paradigm is oversimplified. Macrophages instead exhibit marked heterogeneity and embody a spectrum of activation states that dynamically shift across physiologic conditions (28, 95).

The M1/M2 profile was originally defined from *in vitro* studies in which the M1 macrophage phenotype was described by stimulation with interferon gamma (IFN-γ)/lipopolysaccharide, resulting in expression of inducible nitic oxide synthase and TNF-α, also termed the “classical response”; the M2 phenotype was described by stimulation with IL-4/IL-13, resulting in expression of Arg1/CD206, also termed the “alternative response” (95). Despite this binary characterization, *in vivo* studies that assessed macrophage polarization demonstrated a mixed inflammatory response rather than clear temporal pattern in M1/M2 dichotomy (96). Interestingly, appreciable changes in M1 markers such as inducible nitic oxide synthase or IFN-γ were not observed in nerves for the time points profiled within the first 2 weeks of injury. By contrast, M2 markers such as Arg1, TREM2, and Ym1 increased, with each exhibiting a distinct temporal expression profile during the first 2 weeks after injury (96). Meanwhile, another study examining the temporal dynamics of macrophage polarization during the early injury phase (days 3–5) identified IFN-γ, a proinflammatory (M1-like) cytokine, as a key upstream regulator associated with differentially expressed genes such as Nos2, which were highly expressed at day 5 yet declined over time (97). Cells expressing Arg1 (an M2-like, anti-inflammatory marker) were also identified in which expression decreased from days 5 to 14 but showed a rebound by day 28 (97). Collectively, these initial studies demonstrate the multidimensional complexity of the inflammatory response to nerve injury. Although we maintain M1-like and M2-like terminology for consistency, this strict dichotomy likely does not appreciably represent the plasticity and dynamic states of macrophages (98).

Single-cell sequencing has greatly expanded our understanding of macrophage diversity during homeostasis, injury, and repair states. Notably, it has revealed that multiple macrophage populations coexpress M1/M2 markers across clusters and injury timepoints (71), further reinforcing that this strict dichotomy fails to capture the diverse transcriptional and functional states of macrophages after injury. For example, one study examining nerve crush injury at 1, 3, and 5 days after injury identified about 5 macrophage populations, with the largest attributed to monocyte-derived macrophages expressing Ly6c and Ccr2 (88). Notably, one-to-one matching of clusters across timepoints could not readily be distinguished, underscoring the dynamic macrophage plasticity in response to injury. Furthermore, as described above, multiple groups have now distinguished two resident populations within the nerve: endoneurial-associated macrophages and epineurial-associated macrophages (71, 87, 88, 99). Further examination of each population in response to injury revealed that epineurial macrophages were largely unresponsive to injury, whereas endoneurial macrophages upregulated multiple chemoattractants (Ccl6, Ccl7, Ccl8, Ccl12) between days 1 and 5 after injury (71). To further explore transcriptional diversity as a function of spatial location, Zhao et al. performed scRNA-seq comparing injured vs. distal nerve segments at 3 DPI and identified an Arg1^+^ cluster enriched at the crush injury site, whereas a CD38^+^ population was largely restricted to the distal nerve (88). Complementing this finding, Kalinski et al. employed an Arg1-YFP mouse reporter line and further demonstrated that Arg1-YFP^+^ cells were localized to the crush injury site at 7 DPI but were completely absent from the distal stump, highlighting spatial restriction of specific macrophage subsets (80). Collectively, these studies provide early insight into the spatial organization of macrophage functional states during homeostasis, nerve injury, and recovery.

#### 4.2.4 Recruitment and activation after nerve injury

After the identification that WD is mediated by recruited myeloid cells, critical questions emerged regarding the cues contributing to recruitment and sustained cellular activation. This initial inflammatory response largely involves danger-associated molecular pattern (DAMP) signaling and complement activation (Figure 2).

These respective mechanisms were elaborated by *in vitro* coculturing of nerves with macrophages in the presence of antibodies against complement receptor 3 or C3-deficient serum. Notably, both of these conditions prevented myelin opsonization and phagocytosis (49). *In vivo* complement depletion using cobra venom factor further corroborated these findings; cobra venom factor–treated animals demonstrated significantly lower macrophage numbers, reduced phagocytic activity, and increased myelin retention at 7 DPI (50). Similarly, mice deficient in TLR2, TLR4, or MYD88 had fewer CD68+ macrophages at 7 DPI with correspondingly increased myelin load, demonstrating that DAMP signaling largely drives macrophage recruitment and efferocytosis (47, 49, 50).

Although MCP-1 (CCL2) had long been implicated as a potential macrophage recruitment cue, its role had not been directly tested until work by Siebert et al. (100). In this study, both CCR2 and CCR5 knockout animals were examined to explore the consequences on macrophage recruitment and the sequalae of WD. Notably, CCR2 knockout mice demonstrated lower recruitment and delayed myelin clearance compared with CCR5 knockouts (100). Next, to test the necessity of CCL2 for macrophage recruitment, Siebert and colleagues examined both global and inducible CCL2 knockouts. Surprisingly, both genotypes demonstrated normal macrophage accumulation, foamy macrophage morphology, and effective myelin clearance, suggesting that CCL2 is not uniquely required for recruitment. As CCR2 binds multiple ligands, including CCL7 and CCL12, which were upregulated in the absence of CCL2, these chemokines likely compensate as alternative recruitment cues. Collectively, DAMPs, complement, and chemokines are critical mediators establishing the acute inflammatory microenvironment that drives monocyte/macrophage recruitment during WD (Figure 2). However, further investigation is warranted to elucidate the redundancy and specificity of compensatory ligands and receptors.

#### 4.2.5 Functional roles of macrophages in the injured nerve

Although macrophages were demonstrated to have a central role in WD by phagocytosing myelin and axonal debris, it was not clear whether macrophages also participated in promoting regeneration. Comparative studies between the PNS and CNS (optic nerve) revealed lower macrophage recruitment, scarce evidence of foamy macrophages, and delayed myelin clearance in the CNS, suggesting a fundamental difference in regenerative processes (69). To directly test the necessity of macrophages to PNS regeneration, studies using liposome-mediated macrophage depletion demonstrated reduced macrophage numbers corresponding to delayed myelin degradation (70). Whole-body irradiation experiments to deplete bone marrow myeloid precursors yielded similar results, with reduced myeloid cell recruitment and impaired myelin clearance into the nerve after injury, although potential off-target effects should be noted as a confounding factor (101). An even more targeted approach using CD11b-TK mt-30 mice and ganciclovir administration demonstrated that early myeloid cell ablation severely impaired myelin clearance, neurotrophin synthesis, and vasculogenesis at the injury site (26). Furthermore, by then terminating ganciclovir administration at day 7, there was partial recovery over time, with functional recovery achieved by day 49. Notably, even without early WD, reintroduction of myeloid cells partially restored regenerative processes, although distal myelin debris remained elevated (26).

Macrophages also play a central role in orchestrating the post-injury microenvironment through the secretion of cytokines, chemokines, and trophic factors (Figure 2). Pro-inflammatory cytokines such as TNF, IL-1, and IL-1β recruit additional immune cells and enhance phagocytic function. These cytokines act in an autocrine manner to induce increased macrophage phagocytosis (43) and can instigate neurotrophin production such as nerve growth factor (NGF) to exert positive regenerative effects (102). Furthermore, over the course of WD, there is increased production of anti-inflammatory cytokines (IL-10, IL-6, granulocyte macrophage colony-stimulating factor), with M2-like macrophages serving as a key source of IL-10 and IL-6 (34).

Profiling peripheral nerves after injury has also demonstrated the upregulation of many neurotrophic factors known to promote axonal growth and neuronal survival in both PNS and CNS (Figure 2). Preliminary work demonstrated that addition of neurotrophins to sciatic nerve after injury potentiates axon regeneration, myelination, and functional recovery (103). The cellular origins of NGF were then elaborated by experiments demonstrating that NGF mRNA levels in cultured injured sciatic nerve decreased over time but were rescued with the addition of activated macrophages (103). Furthermore, Barrette et al. (26) examined how depleting CD11b cells impacts neurotrophin synthesis and found that these factors were negligibly detected in the absence of CD11b+ (myeloid) cells. However, this does not rule out the possibility that myeloid cells may communicate with SCs for neurotrophin secretion rather than directly secreting these factors themselves. For example, one study identified that macrophage-derived IL-1β leads to upregulation of NGF from SCs (102). Collectively, macrophages actively contribute to sculpting the post-injury and recovery microenvironment through their secretory functions (26, 103).

Macrophages also actively influence BNB permeability and promote revascularization during nerve regeneration (Figure 2) (104). Assessment of BNB integrity after compression injury revealed greater permeability by 2 weeks, followed by progressive disintegration (105). Depletion of macrophages before injury largely prevented these changes, indicating that macrophages contribute to BNB disruption, although the precise mechanisms remain unknown.

Macrophages also orchestrate post-injury angiogenesis (Figure 2). Early studies observed that axonal regeneration failed in the absence of macrophages, and myeloid cell depletion also reduced blood vessel formation in the distal stump (26). Later work by Cattin et al. (27) demonstrated that revascularization between proximal and distal stumps begins as early as 3 days after injury, forming a bridge across the gap. Co-staining with the SC marker S100 revealed that SCs closely associate with newly forming vessels, migrating along them to extend across the bridge. To further interrogate the mechanisms and cellular species orchestrating angiogenesis, the authors leveraged the knowledge that hypoxia promotes vascular endothelial growth factor (VEGF) secretion, which in turn promotes angiogenesis (27). By using a hypoxia indicator and co-staining with IBA1 to label macrophages, they identified that these cells can sense hypoxic regions, suggesting they secrete VEGF to guide endothelial cells across the bridge. To test this hypothesis, VEGF was selectively depleted in macrophages. Although macrophage numbers at 5 DPI were unchanged, vascularization of the bridge was severely impaired, accompanied by reduced SC migration into the bridge (27). Rescue experiments were also performed in which injection of VEGF into the bridge of knockout animals restored vasculogenesis and enhanced SC migration into the bridge along the newly formed vasculature.

#### 4.2.6 Contribution to inflammatory resolution

Although inflammation is critical to initiate the early injury sequela and promote WD after peripheral nerve injury, persistent inflammation drives pathophysiologic consequences and promotes neurodegeneration (Figure 1). Notably, macrophages participate in inflammatory resolution by efferocytosis (Figure 2) (106, 107). This permits removal of inflammatory molecules such as DAMPs and cytokines from the local environment, transitioning the pro-inflammatory response toward a more anti-inflammatory niche (33, 108). The role of efferocytosis in facilitating WD and favorable regeneration has emerged as an important component of the regenerative process. After nerve injury, macrophages localized to the injury site demonstrate higher expression of efferocytosis-associated genes including MERTK and Gas6 and apoptotic corpse clearance receptors such as TREM2 (80). Notably, a recent study demonstrated that the proportion of macrophages expressing MERTK decreased over time after injury. By using a myeloid cell–specific knockout of MERTK, Pandey et al. observed a reduction of efferocytosis coupled with exacerbated neuropathologic outcomes, suggesting that impaired efferocytosis negatively impacts regenerative potential (109). Furthermore, the type of cell death also influences inflammatory resolution. In sciatic nerve transection, a dysregulated form of macrophage cell death known as pyroptosis skews the cytokine profile toward a more pro-inflammatory signature. By employing gasdermin D knockout mice, a key pyroptosis regulator, this proinflammatory signature was reduced, resulting in improved axonal regeneration and functional outcomes (107). This suggests that when apoptotic cell death is supplanted by pyroptosis, this sustained inflammation adversely impacts the regenerative environment.

Given that the type of cell death impacts inflammatory resolution, it is critical to understand macrophage fate and egress upon WD termination. If the large influx of myeloid cells after injury fails to exit, they may contribute to persistent inflammation and skew regeneration in a pathophysiologic direction. However, the mechanisms underpinning macrophage egress after injury have yielded varied results across injury models (33, 104). Early studies demonstrated that approximately 2%–4% of macrophages undergo apoptosis about 15 days after transection, coinciding with the termination of WD (110, 111). Another study using a transection model and grafting nerves between male and female mice (and vice versa) to distinguish recruited from endogenous monocytes/macrophages revealed that recruited macrophages egress from the graft into perineurial blood vessels, draining lymph nodes, and spleen (110), suggesting most recruited macrophages leave the nerve as inflammation resolves. However, whether the remaining macrophages undergo a different form of cell death remains uninvestigated. Critically, it remains unknown whether egress is maintained in pathophysiologic contexts or if macrophages become trapped and die in failed regenerative environments (33, 104). Therefore, therapeutic interventions promoting efferocytosis and inflammatory resolution present as a viable strategy to promote successful nerve regeneration.

### 4.3 Natural killer cells

Natural killer (NK) cells are innate lymphoid cells traditionally studied for their role in sensing and killing virus-infected cells. However, their contribution to the neuroinflammatory cascade after peripheral nerve injury has remained unexamined until recently. In work by Davies et al. (112), NK cells were identified as infiltrating the sciatic nerve by 3 days after crush injury and increasing in number through day 7 (Figure 2). When NK cells were depleted and nerve injury induced, animals exhibited heightened pain sensitivity. By contrast, by stimulating NK cell activity using IL-2 agonists, greater myelinated axon degeneration and elevated paw withdrawal thresholds were observed, indicating attenuated pain sensitivity. These findings suggest NK cells facilitate myelinated axon degeneration after injury, which translates to reduced pain sensitivity in the acute post-injury phase (112).

Despite this contribution to the neuroimmune narrative, several knowledge gaps persist. Because time points past 7 DPI were not examined, the duration of NK cell persistence in injured nerves and whether accumulation peaks at 7 days after injury remain unknown. Furthermore, IL-2 stimulation was employed to experimentally manipulate and test NK contributions to peripheral nerve injury but may not be physiologically germane to peripheral nerve injury and may have off-target effects on other local cell types. Therefore, the signals regulating NK cell recruitment and activation in the PNS are largely uncharacterized. Given that immune cells demonstrate overlapping and coordinated functions across injury contexts, elucidating cross-talk between NK cells and resident cell types such as perineurial glia and SCs, as well as other recruited immune cells, will be critical for understanding their functional role in shaping peripheral nerve regeneration.

## 5 Adaptive immune response

Adaptive immunity is primarily composed of the response of B cells and T cells to injury and infection. In peripheral nerve injury, interactions between T cells and the innate immune system can regulate, initiate, or prolong inflammation (Figure 2) (113). T cell response to injury is measurable within 15 min, with the adaptive response peaking from 7 to 15 days after injury, followed by a long-term remodeling and memory phase lasting past week 4. Interactions between sympathetic nerve fibers and immune cells can drive inflammatory activation. Cohen et al. determined that optogenetic stimulation of sensory neurons caused TRPV1-mediated release of IL-23 without any external injury, driving CD4+ and γδ T cell expression of IL-17 and initiating an inflammatory response (114). Sympathetic control of immune response can lead to differential T and B cell expression, as shown by a restraint and immunization model examining the effects of acute vs. chronic stress on inflammation (115). Another well-known anti-inflammatory reflex stems from afferent vagus nerve signaling leading to T-cell acetylcholine production (116), and ACh signaling promotes CD4+ T-cell proliferation and T helper response (117). T cells appear to play an important role in neuropathic pain, because their depletion leads to a reduction in pain symptoms and reinsertion into knockout models returns pain measurement to wild-type levels (118). CD4+ T-cell infiltration into injured nerves coincides with development of allodynia, suggesting that T cells play an initiating role (119). Conversely, T helper 2 and regulatory T cell subtypes have been implicated in resolution or reduction in neuropathic pain, emphasizing the importance of phenotype and the dynamic role that T cells play in neuropathic pain and inflammation (120, 121).

B-cell neuroinflammatory and neuropathic pain interactions are primarily driven by various autoimmune interactions. Autoantibodies against voltage-gated potassium channels can cause a range of neuronal hyperexcitability disorders and neuropathic pain, including hyperhiderosis, limbic encephalitis, seizures, psychosis, and gut dysmotility (122). Antibodies against CASPR2 can result in neuromytonia, Morvan syndrome, and neuropathic pain (123). Autoantibodies against citrullinated antigens (such as citrullinated fibrinogen, vimentin, and collagen type II) can induce pain via IgG–FcγR1 interactions and are elevated in patients with rheumatoid arthritis (119). B-cell depletion using anti-CD20 demonstrated the critical role of these cells in the development of allodynia through IgG/immune complex-FcγR interactions (124). These IgG-FcγR were also shown to differentially promote allodynia in a range of peripheral nerve injury models in a dependent fashion on the level of Fc receptor γ-subunit (125). Interestingly, WD appears to be independent of adaptive immune response, as several T- and B-cell knockout models show successful WD and myelin clearance (126), highlighting the interconnected and redundant pathways for the progression and resolution of inflammation in the peripheral nerve.

## 6 Pathophysiologic neuroma formation

Despite the wealth of knowledge demonstrating the role of immune cells in peripheral nerve injury and regeneration, we have a limited understanding of how these responses may be shifted in pathophysiologic contexts (24, 28, 34, 127). Critically, successful regeneration is not the default regenerative program; clinical outcomes predominately result in neuroma-in-continuity manifestation (1), a pathophysiologic form of regeneration hallmarked by propensity of small unmyelinated fibers, fibrosis, and persistent hypercellular state (Figure 1) (15–17). Histology from human samples has revealed that neuromas are persistently inhabited by inflammatory cells including macrophages and adaptive immune cells such as T cells (16). Furthermore, in a basic science model of rapid-stretch nerve injury, we observed pathophysiologic neuroma formation and the coexistence of persistent inflammatory species, similar to human histologic samples (20, 22, 33). The mechanisms underpinning persistent inflammation in neuromas or driving pathophysiologic nerve regeneration remain incompletely defined. Therefore, better understanding and comparing the immune response in successful vs. pathologic regeneration may reveal novel mechanisms amenable for therapeutic intervention. Here, we present two hypotheses to explain why nerve regeneration may be altered and result in pathophysiologic neuroma formation.

### 6.1 Hypothesis 1: failed inflammatory resolution

Failure of inflammatory resolution presents as a potential candidate precipitating pathophysiologic neuroma formation. Pro-inflammatory microenvironments that promote incendiary forms of cell death, such as NETosis for neutrophils or pyroptosis for macrophages, may potentiate a self-amplifying cycle of inflammation. Failure of resolution also contributes to exacerbated fibrosis (108, 128), a hallmark of neuromas often ascribed to inhibiting axonal regeneration (129). Furthermore, in our neuroma-forming injury model, we observed CD11b+ immune cells restricted to neuroma borders, accompanied by pronounced hypercellularity but reduced nuclear staining centrally, suggesting a necrotic core within the heart of the neuroma (104). However, neither cell death pathways nor resolution mechanisms have been investigated in the context of neuroma formation. Collectively, the hypercellularity and persistent inflammation in both human neuroma specimens (16) and basic science models of neuroma formation (22, 32, 33) suggest that mechanistic studies of cell death cascades and inflammatory resolution warrant further investigation.

### 6.2 Hypothesis 2: neuroimmune signaling sustains inflammatory states

Another potential mechanism precipitating neuroma formation involves the bidirectional communication between neurons and inflammatory cells. For example, nociceptors are increasingly recognized for their role in orchestrating inflammatory responses, with growing evidence highlighting neuron–immune cell interactions as part of the “sensory neuroimmune frontier” (130, 131). Through the secretion of neuropeptides such as calcitonin gene-related peptide (CGRP) and substance P, nociceptors can modulate pain and shape inflammatory responses (132, 133). For example, CGRP derived from nociceptors promotes wound healing by acting on neutrophils and macrophages to enhance efferocytosis and direct macrophages toward repair phenotypes (133). Furthermore, nociceptors can also mediate inflammatory effects in response to pathogens: for example, *Staphylococcus aureus* directly activates nociceptors, triggering calcitonin gene-related peptide release and resulting in a dampened pro-inflammatory immune response (130). Although evidence supports the interaction of nociceptors both promoting or modulating inflammation, this interaction is likely context specific. For example, in severe nerve injuries in which axonal endings are exposed to the microenvironment early after injury, nerve fibers may promote persistent inflammation and excessive fibrotic deposition associated with neuroma formation.

## 7 Therapeutic interventions

**Figure 3:**
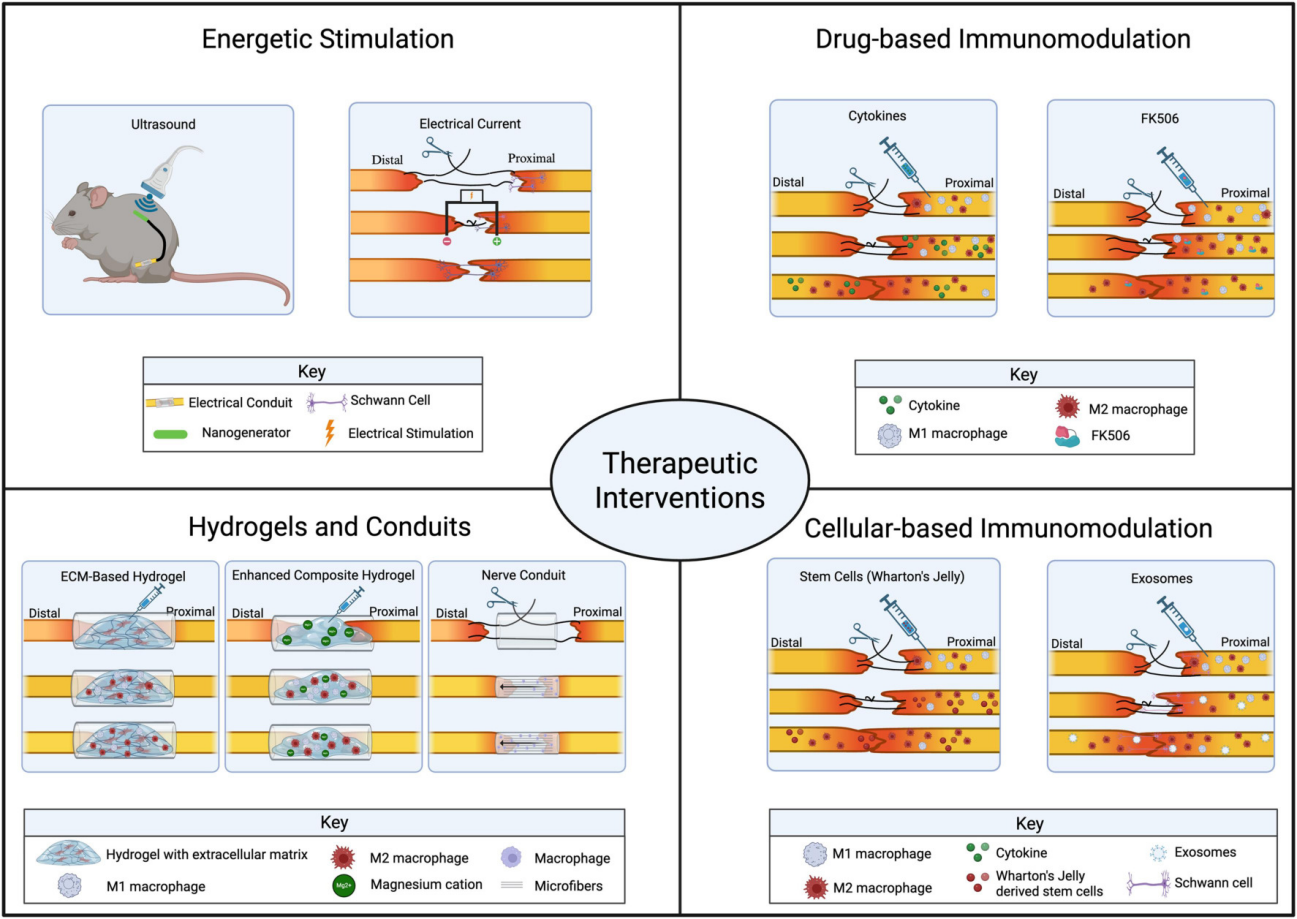
Overview of four major categories of therapeutic approaches for peripheral nerve regeneration. Energetic stimulation (top left): Ultrasound stimulation applied to the injury site and electrical current applied across the nerve gap. Drug-based immunomodulation (top right): Delivery of cytokines or FK506 to modulate the immune response. Hydrogels and conduits (bottom left): Extracellular matrix–based hydrogel scaffolds, enhanced composite hydrogels incorporating macrophages and magnesium cations (Mg²^+^), and nerve conduits with aligned microfibers for axonal guidance. Cellular-based immunomodulation (bottom right): Transplantation of Wharton's jelly-derived stem cells or exosomes. Figure created with BioRender.com.

Traditional strategies for nerve repair have focused on restoring continuity across the injury site and facilitating reapproximation of nerve endings by providing structural support and axonal guidance using biocompatible scaffolds (Figure 3) (134). However, insights from basic science have demonstrated that inflammation is both critical to successful nerve regeneration and potentially pathologic when aberrantly activated, thereby prompting a shift in biomaterial design strategy (135). Innovative approaches now aim to promote regeneration by modulating the immune microenvironment. Here, we examine immunomodulation strategies including drug-based and cellular therapies, energetic stimulation, hydrogels, and conduits that optimize nerve regeneration.

### 7.1 Drug-based immunomodulation

#### 7.1.1 Anti-inflammatory treatments

Anti-inflammatory treatments focus on reducing the acute and chronic inflammatory milieu that impairs axonal regrowth in the injured sciatic nerve (Figure 3). For example, FK506 reduces levels of pro-inflammatory cytokines such as IL-1β and TNF-α, modulates macrophage phenotype from pro-inflammatory to anti-inflammatory, and inhibits ECM remodeling enzymes like MMP13 (136). These effects collectively decrease nerve edema and macrophage infiltration, thereby fostering a more permissive microenvironment for regeneration. Other anti-inflammatory agents, including nonsteroidal anti-inflammatory drugs and minocycline, also demonstrate efficacy in reducing macrophage-mediated neurotoxicity and scar formation, although their specific effects in peripheral nerve injury models require further detailed studies (137, 138).

#### 7.1.2 Immunomodulatory treatments

Cytokines and growth factors serve as critical mediators in the neuroimmune crosstalk during peripheral nerve regeneration (Figure 3). Therapeutic administration of cytokines such as IL-33 and IL-4 promotes the polarization of macrophages toward a M2-like phenotype, characterized by secretion of anti-inflammatory cytokines like IL-10 and enhanced production of neurotrophic factors including NGF, brain-derived neurotrophic factor (BDNF), and VEGF (136). This shift attenuates inflammation while supporting SC activity and axonal regrowth. Growth factors such as TGF-β1 further regulate immune responses via Smad-dependent signaling and contribute to ECM remodeling, myelination, and reduction of neuropathic pain (139).

### 7.2 Cellular therapies

Stem cell-based therapies leverage both the immunomodulatory and regenerative potential of mesenchymal stem cells, neural stem cells, and their secreted exosomes to enhance peripheral nerve repair (Figure 3). Mesenchymal stem cells derived from sources such as Wharton's jelly have been shown to exert anti-inflammatory effects by promoting regulatory T cell induction and enhancing cytokine secretion, which suppress detrimental immune responses (140). Additionally, neural stem cell–conditioned media have been shown to inhibit macrophage infiltration and downregulate pro-inflammatory cytokine expression through pathways such as Sirt-1 (141). Exosomes derived from induced pluripotent stem cells promote SC proliferation, migration, and myelination by activating intracellular signaling pathways such as PI3K-AKT and focal adhesion kinase (142). These therapies provide a multimodal approach that combines immune modulation with direct trophic support to neurons and glia, facilitating both structural and functional recovery.

### 7.3 Energetic stimulation

External stimulation has demonstrated improvements in nerve regeneration through various mechanisms, with the most promising treatments including electrical current, ultrasound, laser therapy, and magnetic fields (Figure 3) (143). Electrical stimulation is commonly used for improving nerve regeneration outcomes, demonstrating improved proliferation and function of SCs, fibroblasts, and neural cells within a nerve injury (144). Electrical stimulation improves SC and neurite guidance in the direction of the applied electric field (145) and increases production of factors such as NGF, BDNF, and glial cell line–derived neurotrophic factor (146). Electrical stimulation also appears to reduce inflammation through inhibition of neuronal release of inflammatory factors such as CGRP, high mobility group box 1 protein, and substance P (147), as well as improved angiogenesis and vascular ingrowth through increased production of VEGF (28). Low-intensity pulsed ultrasound has been shown to increase SC production of NGF, resulting in thicker myelin regrowth and better nerve gap crossing (148, 149). Low-level laser therapy is another promising method for improving nerve injury outcomes and has demonstrated improved SC proliferation and expression of neurotrophic factors (150), as well as reduction in free-radical production and macrophage infiltration (151). Laser therapy has the advantages of highly specific targeting and deep tissue penetration for near-infrared wavelengths. Finally, magnetic stimulation has shown promise in nerve regeneration improvement through similar mechanisms as electrical stimulation via induced current and axon membrane depolarization. Magnetic stimulation has improved nerve–muscle cross-talk, increased neuromuscular junction maturation (152), and enhanced production of neurotrophic factors (153).

### 7.4 Hydrogel-based immunomodulatory strategies

Hydrogels are largely composed of water and polymeric networks and derived from decellularized porcine ECM for peripheral nerve injury applications (154). Because guiding scaffolds appear to be necessary for nerve regeneration, traditional strategies have employed hydrogels composed of endogenous matrix components such as collagen and laminin combined with growth factors to promote axonal regeneration (134). Furthermore, hydrogels offer advantages over traditional rigid scaffolds because of their tunable properties and ability to adapt to dynamic changes in the injury microenvironment. Recent studies now focus on how hydrogel composition affects immune responses and the potential for hydrogels themselves to sculpt the immune microenvironment. Two classes of hydrogels have been investigated: those using ECM-based approaches and those using enhanced composite designs (Figure 3).

#### 7.4.1 ECM-based approaches

Decellularized xenogeneic hydrogels have demonstrated immunomodulatory effects across multiple injury models. In a 15-mm nerve gap transection model with silicon conduits filled with decellularized xenogeneic hydrogels, macrophages were found to dominate the graft margins and migrate further into the conduit compared with controls without hydrogel (155). Although spatial distribution patterns of each were not fully described, there also appeared to be greater recruitment of M2 macrophages in the hydrogel-infused conduit, resulting in a greater M2:M1 ratio. In a shorter gap model at 5 days after injury, the hydrogel treatment showed decreased expression of genes associated with inflammation and scarring, such as collagens and fibroblast growth factor receptor, whereas genes associated with an anti-inflammatory profile were increased compared with conduit-only controls (156). At 10 days after injury, the hydrogel treatment group also demonstrated a decrease in M1-like macrophages but no observable differences in M2-like macrophages, resulting in a higher M2:M1 ratio. Furthermore, conduits with hydrogel matrix showed significantly more neuronal cell bodies labeled with retrograde tracer, demonstrating increased axonal crossing associated with the higher M2:M1 ratio (156). Finally, in a nerve crush-injured model, injection of a xenogeneic decellularized nerve matrix reduced M1-like macrophages at both 3 and 7 days after injury, corroborated by decreased pro-inflammatory cytokine expression (TNF-α and IL-1β) (154). Conversely, more M2-like macrophages and anti-inflammatory cytokines (TGF-β and IL-10) were observed in the hydrogel-treated condition. Western blot revealed higher expression of TLR4/MyD88/NF-κB axis proteins in the untreated group, suggesting mechanistic involvement of this signaling pathway in the anti-inflammatory effects.

#### 7.4.2 Enhanced composite hydrogels

Although decellularized xenograft hydrogels are the most common strategy employed, hydrogels with alternative compositions have been developed (Figure 3). For example, silk fibroin derived from silkworm cocoons offers low immunogenicity, presenting as a potential biocompatible and natural biomaterial for hydrogel composition. By enriching hydrogels with Mg²^+^ and silk fibroin, an increased M2:M1 ratio was observed compared with untreated NH4 commercial chitosan conduit groups at both 7 and 14 days after injury (157). Others have attempted to harness bioelectrical properties in conjunction with decellularized ECM to create conductive, biodegradable scaffolds to enhance nerve regeneration. In a 4-week post-transection gap model, scaffolds incorporating polydopamine-modified silicon phosphorus nanosheets to enhance conductivity exhibited reduced pro-inflammatory and increased anti-inflammatory macrophage densities (158). Collectively, these studies demonstrate that hydrogel composition can modulate macrophage phenotype and improve axon regeneration. Further investigation into the effects on other glial types such as perineurial glia and SCs, recruited immune cells, revascularization, and ECM remodeling, as well as comprehensive evaluation of functional outcomes, will be critical for clinical translation.

### 7.5 Nerve conduits and physical cue-mediated immune regulation

Biochemical properties are critical for modulating immune responses, but growing evidence suggests that structural and mechanical properties can also impact immune cell polarization profiles (159). Foremost, cellular growth and migration are greatly impacted by material orientation (160). Cells grown *in vitro* on randomly oriented microstructures adopt abnormal, oblong phenotypes and grow in a haphazard pattern matching the material orientation. This knowledge can be leveraged in the incorporation of microfibers inserted into conduits to facilitate cellular migration, allowing cells to crawl along the structure and use aligned fibers as scaffolds (Figure 3). In a moderate-gap (11-mm) injury model, conduits with aligned fibers promoted elongated morphology and parallel arrangement of both macrophages and SCs along the proximal-to-distal axis compared with cells in disordered conduits (161). These conduits also exhibited more macrophage recruitment or proliferation at 7 dpi, with higher total macrophage numbers in the aligned microfiber group. However, the number of M1-like macrophages decreased relative to randomly arranged microfiber groups, while M2-like macrophages were increased. Jia et al. observed similar outcomes at a longer time point (21 days) after injury (162). Although total macrophage numbers were not statistically significantly different between aligned and randomly oriented fiber groups, a higher percentage of macrophages in the aligned group adopted an M2-like, anti-inflammatory phenotype. Therefore, providing an internal scaffold to guide cellular migration into the conduit with orientation mirroring native nerve structure appears to impact immune cell polarization toward a more anti-inflammatory phenotype.

Attention to electrical and mechanical properties of conduits is also gaining heightened appreciation. In one study, electroactive aniline trimer-based polyurethane was added to conduits to improve conduction (163). *In vitro* assessment of cells seeded on electroactive conduits demonstrated increased mRNA expression of pro-regenerative genes such as Arg1 and decreased expression of pro-inflammatory genes such as Nos2. Subsequently, increased anti-inflammatory cytokines (IL-10) and decreased pro-inflammatory cytokines (TNF-α) were also observed. Another study focused on matching conduit mechanical properties (e.g., stiffness) to the nerve tissue (164). Greater stiffness may result in nerve entrapment or hamper regeneration, whereas low elastic modulus may result in collapse after transplantation and subsequent compression. By matching the elastic modulus of the conduit to the native peripheral nerve tissue, there is potential to mitigate physical challenges of conduit transplantation. Notably, leveraging this mechanically matched strategy resulted in elevated gene and protein expression of anti-inflammatory markers and M2-like macrophages (164). Therefore, considering how material properties of both hydrogels and conduits impact inflammatory responses is a critical step in advancing translatable strategies to improve nerve regeneration.

### 7.6 Translational challenges

Substantial progress has been made in identifying the spatial and temporal immune response to peripheral nerve injury. Furthermore, these basic science insights provide the foundation for the development of translational interventions to promote favorable regeneration. However, despite this progress, we still lack approved therapeutic interventions that can be used in clinical settings to promote peripheral nerve regeneration.

#### 7.6.1 Global challenges

As in other biomedical modalities, species-specific differences may explain challenges to translation in peripheral nerve repair (165, 166). For example, most experimental models use rodents, whose nerves regenerate faster than human nerves and are uni- or bifascicular rather than polyfascicular, as in humans (167). In addition, most research studies are performed on male animals. Although this mirrors the human demographic in which these injuries predominately occur, there likely exist sex-specific differences in regeneration outcomes that could provide valuable insight for the development of therapeutic interventions (168). Furthermore, peripheral nerve injuries often lead to chronic neuropathic pain, and sex-specific differences in pain processing and pathophysiology are well documented (169). Finally, most preclinical studies fail to approximate the complex, highly variable, real-world clinical scenario. For example, animal studies commonly utilize short-gap injuries with immediate intervention, whereas clinical repairs often involve more complex injuries with delayed treatment timelines, creating temporal mismatches that complicate direct translation (170, 171). Another critical challenge is the difficulty of translating basic science outcome measures to clinically meaningful endpoints. It is not clear how certain outcomes indicative of recovery in preclinical models, such as the number of axons regenerating, amount of reinnervation, or electrophysiologic outcomes, directly correlate to neurologic improvement in patients (172). Establishing clinically meaningful endpoints that predict functional recovery remains a significant barrier to assessing therapeutic efficacy.

#### 7.6.2 Drug-based and cellular therapies

For drug-based therapies, a critical challenge is cell-specific targeting. Challenges with drug-based application of agents such as growth factors or immunosuppressive agents include ideal dosing strategies and temporal regimes (165). Currently, many interventions target receptors or pathways with inherent multiplicity across multiple cell types, and beneficial outcomes may be offset by negative side effects (173). Cellular-based strategies face challenges including immunogenicity, poor cell survival rates that can exacerbate local inflammation, and costs associated with patient-specific manufacturing that limit accessibility (140, 174). Finally, given the dynamic microenvironment of peripheral nerve regeneration, we still lack understanding of temporal and spatial changes to cell phenotypes and their synergistic interactions. Global strategies to alter regeneration are likely insufficient, and we need improved temporal and spatial understanding of favorable and failed regeneration to develop targeted interventions (28).

#### 7.6.3 Electrical stimulation

In the realm of electrical stimulation, one of the critical challenges is the optimization of stimulation protocols from rodent to human, or for specific neuronal types; however, there is promising evidence of therapeutic benefits in several preclinical models. Notably, electrical stimulation has been shown to improve regeneration through increased expression of growth factors associated with regeneration, such as NGF and BDNF, which are associated with improved axon regeneration and motor reinnervation (146, 175). Despite these benefits, not much is known about how electrical stimulation may modulate the immune microenvironment. Further interrogation of the impacts of electrical stimulation not only on nerves themselves but also on other cells such as fibroblasts, glia, regenerating vasculature, and infiltrating immune cells will be critical to optimize protocols for temporal and potentially broader improvement in the local microenvironment.

In addition, there is need for more studies in humans to establish standard protocols, outcome measures, and demonstrate safety for broader applicability (176). Although promising results exist in both rodent and human studies across diverse injury mechanisms and nerves, the expansion of this approach remains limited. This is largely a consequence of substantial variability among studies, including stimulation protocols, injury paradigms, timing of intervention after injury, and the use of direct nerve stimulation vs. transcutaneous paradigms during recovery (176), as well as marginal and inconsistent outcomes. Electrical stimulation likely follows a dose-response curve, but there are numerous factors that determine dosage. As of yet, there is no clear determination of optimal dosage.

#### 7.6.4 Conduits and hydrogels

For conduits, a critical challenge is the identification of materials that minimize immunogenicity while providing ideal mechanical properties to match native nerve tissue (162, 164). Furthermore, the gap size in mice and humans differs markedly. Whereas most preclinical studies use short gaps, clinical gaps often exceed these distances, and vascularization and cellular infiltration kinetics differ fundamentally (177, 178).

Despite the wide variety and novel insights into how conduits and hydrogels not only facilitate reapproximation but can also be leveraged with molecules that facilitate regeneration or bias infiltrating or local immune and glial cells toward pro-regenerative phenotypes, very few grafts have been approved for clinical use (165). Moreover, several technical challenges still exist, such as prolific axonal branching at the site of repair and increased axonal sprouting at axon terminals, resulting in polyinnervation of muscles (165). No conduit or hydrogel system has successfully bridged large gaps with functional outcomes comparable with autograft (171).

## 8 Conclusion

Despite the capacity for PNS regeneration, clinical outcomes remain poor and are hallmarked by the development of comorbidities such as chronic pain, depression, and anxiety. The immune system is fundamental in coordinating a microenvironment conducive to successful regeneration. However, in clinical contexts, this regenerative program is undermined, resulting instead in pathophysiologic neuroma formation. Recent advances have expanded our understanding of the inflammatory response during successful regeneration, but critical questions remain regarding how these pathways become maladaptive in the pathophysiologic state. Bridging mechanistic insights from experimental models with observations from human tissues will be essential to define how inflammation can be therapeutically leveraged to orchestrate successful regeneration. Coordinated efforts between scientists and clinicians to further elaborate the immune response to peripheral nerve injury will be cardinal for translating mechanistic insights into effective therapeutic interventions that improve patient outcomes.

## References

[1] Lundborg G. A 25-year perspective of peripheral nerve surgery: evolving neuroscientific concepts and clinical significance. J Hand Surg Am. (2000) 25(3):391–414. 10.1053/jhsu.2000.4165 10811744

[2] Rasulić L Savić A Živković B Vitošević F Mićović M Baščarević V Outcome after brachial plexus injury surgery and impact on quality of life. Acta Neurochir. (2017) 159(7):1257–64. 10.1007/s00701-017-3205-1 28540442

[3] Nicholson B Verma S. Comorbidities in chronic neuropathic pain. Pain Med. (2004) 5(Suppl 1):S9–27. 10.1111/j.1526-4637.2004.04019.x 14996227

[4] Foster CH Karsy M Jensen MR Guan J Mahan EI Mahan MA. Trends and cost-analysis of lower extremity nerve injury using the national inpatient sample. Neurosurgery. (2019) 85(2):250–6. 10.1093/neuros/nyy265 29889258

[5] Karsy M Watkins R Jensen MR Guan J Brock AA Mahan MA. Trends and cost analysis of upper extremity nerve injury using the national (nationwide) inpatient sample. World Neurosurg. (2019) 123:e488–500. 10.1016/j.wneu.2018.11.192 30502477

[6] Midha R. Epidemiology of brachial plexus injuries in a multitrauma population. Neurosurgery. (1997) 40(6):1182–9; discussion 8–9. 10.1097/00006123-199706000-00014 9179891

[7] Kaiser R Waldauf P Haninec P. Types and severity of operated supraclavicular brachial plexus injuries caused by traffic accidents. Acta Neurochir. (2012) 154(7):1293–7. 10.1007/s00701-012-1291-7 22302237

[8] Kaiser R Waldauf P Ullas G Krajcová A. Epidemiology, etiology, and types of severe adult brachial plexus injuries requiring surgical repair: systematic review and meta-analysis. Neurosurg Rev. (2020) 43(2):443–52. 10.1007/s10143-018-1009-2 30014280

[9] Kim DH Cho YJ Tiel RL Kline DG. Outcomes of surgery in 1019 brachial plexus lesions treated at Louisiana State University Health Sciences Center. J Neurosurg. (2003) 98(5):1005–16. 10.3171/jns.2003.98.5.1005 12744360

[10] Choi PD Novak CB Mackinnon SE Kline DG. Quality of life and functional outcome following brachial plexus injury. J Hand Surg Am. (1997) 22(4):605–12. 10.1016/S0363-5023(97)80116-5 9260614

[11] Varadarajan SG Hunyara JL Hamilton NR Kolodkin AL Huberman AD. Central nervous system regeneration. Cell. (2022) 185(1):77–94. 10.1016/j.cell.2021.10.029 34995518 PMC10896592

[12] Mahar M Cavalli V. Intrinsic mechanisms of neuronal axon regeneration. Nat Rev Neurosci. (2018) 19(6):323–37. 10.1038/s41583-018-0001-8 29666508 PMC5987780

[13] Dubuisson AS Kline DG. Brachial plexus injury: a survey of 100 consecutive cases from a single service. Neurosurgery. (2002) 51(3):673–83; discussion 82–3. 10.1097/00006123-200209000-00011 12188945

[14] Kline DG. Surgical repair of peripheral nerve injury. Muscle Nerve. (1990) 13(9):843–52. 10.1002/mus.880130911 2233871

[15] Cravioto H Battista A. Clinical and ultrastructural study of painful neuroma. Neurosurgery. (1981) 8(2):181–90. 10.1227/00006123-198102000-00007 7207783

[16] Mahan MA Abou-Al-Shaar H Karsy M Warner W Yeoh S Palmer CA. Pathologic remodeling in human neuromas: insights from clinical specimens. Acta Neurochir. (2019) 161(12):2453–66. 10.1007/s00701-019-04052-7 31612277

[17] Chen L Gao SC Gu YD Hu SN Xu L Huang YG. Histopathologic study of the neuroma-in-continuity in obstetric brachial plexus palsy. Plast Reconstr Surg. (2008) 121(6):2046–54. 10.1097/PRS.0b013e3181706e7e 18520895

[18] McLean NA Verge VMK. Dynamic impact of brief electrical nerve stimulation on the neural immune axis-polarization of macrophages toward a pro-repair phenotype in demyelinated peripheral nerve. Glia. (2016) 64(9):1546–61. 10.1002/glia.23021 27353566

[19] Woodhall B. Modern history of peripheral nerve surgery; world war II and the postwar study of peripheral nerve regeneration. J Am Med Assoc. (1949) 139(9):564–6. 10.1001/jama.1949.02900260010003 18109438

[20] Mahan MA Yeoh S Monson K Light A. Rapid stretch injury to peripheral nerves: biomechanical results. Neurosurgery. (2019) 85(1):E137–44. 10.1093/neuros/nyy423 30383240

[21] Warner WS Yeoh S Light A Zhang J Mahan MA. Rapid-stretch injury to peripheral nerves: histologic results. Neurosurgery. (2020) 86(3):437–45. 10.1093/neuros/nyz194 31140562

[22] Mahan MA Warner WS Yeoh S Light A. Rapid-stretch injury to peripheral nerves: implications from an animal model. J Neurosurg. (2020) 133(5):1537–47. 10.3171/2019.6.JNS19511 31585426

[23] Avellino AM Hart D Dailey AT MacKinnon M Ellegala D Kliot M. Differential macrophage responses in the peripheral and central nervous system during Wallerian degeneration of axons. Exp Neurol. (1995) 136(2):183–98. 10.1006/exnr.1995.1095 7498408

[24] Fu SY Gordon T. The cellular and molecular basis of peripheral nerve regeneration. Mol Neurobiol. (1997) 14(1–2):67–116. 10.1007/BF02740621 9170101

[25] Waller A. Experiments on the section of the glosso-pharyngeal and hypoglossal nerves of the frog, and observations of the alterations produced thereby in the structure of their primitive fibres. Edinb Med Surg J. (1851) 76(189):369–76. 30332247 PMC5929074

[26] Barrette B Hébert MA Filali M Lafortune K Vallières N Gowing G Requirement of myeloid cells for axon regeneration. J Neurosci. (2008) 28(38):9363–76. 10.1523/JNEUROSCI.1447-08.2008 18799670 PMC6671109

[27] Cattin AL Burden JJ Van Emmenis L Mackenzie FE Hoving JJ Garcia Calavia N Macrophage-induced blood vessels guide Schwann cell-mediated regeneration of peripheral nerves. Cell. (2015) 162(5):1127–39. 10.1016/j.cell.2015.07.021 26279190 PMC4553238

[28] Cattin AL Lloyd AC. The multicellular complexity of peripheral nerve regeneration. Curr Opin Neurobiol. (2016) 39:38–46. 10.1016/j.conb.2016.04.005 27128880

[29] Glass CK Saijo K Winner B Marchetto MC Gage FH. Mechanisms underlying inflammation in neurodegeneration. Cell. (2010) 140(6):918–34. 10.1016/j.cell.2010.02.016 20303880 PMC2873093

[30] Goswami KK Bose A Baral R. Macrophages in tumor: an inflammatory perspective. Clin Immunol. (2021) 232:108875. 10.1016/j.clim.2021.108875 34740843

[31] Vora AR Bodell SM Loescher AR Smith KG Robinson PP Boissonade FM. Inflammatory cell accumulation in traumatic neuromas of the human lingual nerve. Arch Oral Biol. (2007) 52(1):74–82. 10.1016/j.archoralbio.2006.08.015 17097599

[32] Yeoh S Warner WS Eli I Mahan MA. Rapid-stretch injury to peripheral nerves: comparison of injury models. J Neurosurg. (2021) 135(3):893–903. 10.3171/2020.5.JNS193448 33157535

[33] Warner WS Stubben C Yeoh S Light AR Mahan MA. Next-generation RNA sequencing elucidates transcriptomic signatures of pathophysiologic nerve regeneration. Sci Rep. (2023) 13(1):8856. 10.1038/s41598-023-35606-6 37258605 PMC10232541

[34] Gaudet AD Popovich PG Ramer MS. Wallerian degeneration: gaining perspective on inflammatory events after peripheral nerve injury. J Neuroinflammation. (2011) 8:110. 10.1186/1742-2094-8-110 21878126 PMC3180276

[35] Guo Q Zhu H Wang H Zhang P Wang S Sun Z Transcriptomic landscapes of immune response and axonal regeneration by integrative analysis of molecular pathways and interactive networks post-sciatic nerve transection. Front Neurosci. (2018) 12:457. 10.3389/fnins.2018.00457 30038556 PMC6046400

[36] He B Pang V Liu X Xu S Zhang Y Djuanda D Interactions among nerve regeneration, angiogenesis, and the immune response immediately after sciatic nerve crush injury in Sprague-Dawley rats. Front Cell Neurosci. (2021) 15:717209. 10.3389/fncel.2021.717209 34671243 PMC8522912

[37] Murtazina A Adameyko I. The peripheral nervous system. Development. (2023) 150(9):dev201164. 10.1242/dev.201164 37170957

[38] Kucenas S. Perineurial glia. Cold Spring Harb Perspect Biol. (2015) 7(6):a020511. 10.1101/cshperspect.a020511 25818566 PMC4448606

[39] Peltonen S Alanne M Peltonen J. Barriers of the peripheral nerve. Tissue Barriers. (2013) 1(3):e24956. 10.4161/tisb.24956 24665400 PMC3867511

[40] Ubogu EE. Biology of the human blood-nerve barrier in health and disease. Exp Neurol. (2020) 328:113272. 10.1016/j.expneurol.2020.113272 32142802 PMC7145763

[41] Constantin AM Boşca AB Crivii CB Crintea A Sufleţel RT Alexandru BC The intriguing perineurial cells—an updated overview of their origin, structure, functions and implication in pathology. Rom J Morphol Embryol. (2024) 65(4):567–74. 10.47162/RJME.65.4.02 39957017 PMC11924920

[42] Palladino SP Helton ES Jain P Dong C Crowley MR Crossman DK The human blood-nerve barrier transcriptome. Sci Rep. (2017) 7(1):17477. 10.1038/s41598-017-17475-y 29234067 PMC5727190

[43] Shamash S Reichert F Rotshenker S. The cytokine network of Wallerian degeneration: tumor necrosis factor-alpha, interleukin-1alpha, and interleukin-1beta. J Neurosci. (2002) 22(8):3052–60. 10.1523/JNEUROSCI.22-08-03052.2002 11943808 PMC6757534

[44] Perrin FE Lacroix S Aviles-Trigueros M David S. Involvement of monocyte chemoattractant protein-1, macrophage inflammatory protein-1alpha and interleukin-1beta in Wallerian degeneration. Brain. (2005) 128(Pt 4):854–66. 10.1093/brain/awh407 15689362

[45] Tofaris GK Patterson PH Jessen KR Mirsky R. Denervated Schwann cells attract macrophages by secretion of leukemia inhibitory factor (LIF) and monocyte chemoattractant protein-1 in a process regulated by interleukin-6 and LIF. J Neurosci. (2002) 22(15):6696–703. 10.1523/JNEUROSCI.22-15-06696.2002 12151548 PMC6758146

[46] Takeda K Kaisho T Akira S. Toll-like receptors. Annu Rev Immunol. (2003) 21:335–76. 10.1146/annurev.immunol.21.120601.141126 12524386

[47] Boivin A Pineau I Barrette B Filali M Vallières N Rivest S Toll-like receptor signaling is critical for Wallerian degeneration and functional recovery after peripheral nerve injury. J Neurosci. (2007) 27(46):12565–76. 10.1523/JNEUROSCI.3027-07.2007 18003835 PMC6673340

[48] Ramaglia V King RHM Nourallah M Wolterman R de Jonge R Ramkema M The membrane attack complex of the complement system is essential for rapid Wallerian degeneration. J Neurosci. (2007) 27(29):7663–72. 10.1523/JNEUROSCI.5623-06.2007 17634361 PMC6672891

[49] Brück W Friede RL. The role of complement in myelin phagocytosis during PNS Wallerian degeneration. J Neurol Sci. (1991) 103(2):182–7. 10.1016/0022-510X(91)90162-Z 1880536

[50] Dailey AT Avellino AM Benthem L Silver J Kliot M. Complement depletion reduces macrophage infiltration and activation during Wallerian degeneration and axonal regeneration. J Neurosci. (1998) 18(17):6713–22. 10.1523/JNEUROSCI.18-17-06713.1998 9712643 PMC6792968

[51] Liu L Lioudyno M Tao R Eriksson P Svensson M Aldskogius H. Hereditary absence of complement C5 in adult mice influences Wallerian degeneration, but not retrograde responses, following injury to peripheral nerve. J Peripher Nerv Syst. (1999) 4(2):123–33. 10442688

[52] Einheber S Hannocks MJ Metz CN Rifkin DB Salzer JL. Transforming growth factor-beta 1 regulates axon/Schwann cell interactions. J Cell Biol. (1995) 129(2):443–58. 10.1083/jcb.129.2.443 7536747 PMC2199906

[53] Fregnan F Muratori L Simoes AR Giacobini-Robecchi MG Raimondo S. Role of inflammatory cytokines in peripheral nerve injury. Neural Regen Res. (2012) 7(29):2259–66. 10.3969/j.issn.1673-5374.2012.29.003 25538747 PMC4268726

[54] Vougioukas VI Roeske S Michel U Bruck W. Wallerian degeneration in ICAM-1-deficient mice. Am J Pathol. (1998) 152(1):241–9. 9422541 PMC1858117

[55] Liu Q Wang X Yi S. Pathophysiological changes of physical barriers of peripheral nerves after injury. Front Neurosci. (2018) 12:597. 10.3389/fnins.2018.00597 30210280 PMC6119778

[56] Liao S Chen Y Luo Y Zhang M Min J. The phenotypic changes of Schwann cells promote the functional repair of nerve injury. Neuropeptides. (2024) 106:102438. 10.1016/j.npep.2024.102438 38749170

[57] Jessen KR Mirsky R. The repair Schwann cell and its function in regenerating nerves. J Physiol. (2016) 594(13):3521–31. 10.1113/JP270874 26864683 PMC4929314

[58] Höke A Redett R Hameed H Jari R Zhou C Li ZB Schwann cells express motor and sensory phenotypes that regulate axon regeneration. J Neurosci. (2006) 26(38):9646–55. 10.1523/JNEUROSCI.1620-06.2006 16988035 PMC6674436

[59] Harty BL Monk KR. Unwrapping the unappreciated: recent progress in Remak Schwann cell biology. Curr Opin Neurobiol. (2017) 47:131–7. 10.1016/j.conb.2017.10.003 29096241 PMC5963510

[60] Nocera G Jacob C. Mechanisms of Schwann cell plasticity involved in peripheral nerve repair after injury. Cell Mol Life Sci. (2020) 77(20):3977–89. 10.1007/s00018-020-03516-9 32277262 PMC7532964

[61] Jiang M Chen M Liu N. Interactions between Schwann cell and extracellular matrix in peripheral nerve regeneration. Front Neurol. (2024) 15:1372168. 10.3389/fneur.2024.1372168 38651098 PMC11034552

[62] Jessen KR Mirsky R Lloyd AC. Schwann cells: development and role in nerve repair. Cold Spring Harb Perspect Biol. (2015) 7(7):a020487. 10.1101/cshperspect.a020487 25957303 PMC4484967

[63] Nave KA Werner HB. Ensheathment and myelination of axons: evolution of glial functions. Annu Rev Neurosci. (2021) 44:197–219. 10.1146/annurev-neuro-100120-122621 33722070

[64] Lewis GM Kucenas S. Perineurial glia are essential for motor axon regrowth following nerve injury. J Neurosci. (2014) 34(38):12762–77. 10.1523/JNEUROSCI.1906-14.2014 25232113 PMC4166161

[65] Arena KA Zhu Y Kucenas S. Transforming growth factor-beta signaling modulates perineurial glial bridging following peripheral spinal motor nerve injury in zebrafish. Glia. (2022) 70(10):1826–49. 10.1002/glia.24220 35616185 PMC9378448

[66] Schröder JM May R Weis J. Perineurial cells are the first to traverse gaps of peripheral nerves in silicone tubes. Clin Neurol Neurosurg. (1993) 95(Suppl):S78–83. 10.1016/0303-8467(93)90040-N 8467601

[67] Perkins NM Tracey DJ. Hyperalgesia due to nerve injury: role of neutrophils. Neuroscience. (2000) 101(3):745–57. 10.1016/S0306-4522(00)00396-1 11113323

[68] Nadeau S Filali M Zhang J Kerr BJ Rivest S Soulet D Functional recovery after peripheral nerve injury is dependent on the pro-inflammatory cytokines IL-1beta and TNF: implications for neuropathic pain. J Neurosci. (2011) 31(35):12533–42. 10.1523/JNEUROSCI.2840-11.2011 21880915 PMC6703268

[69] Perry VH Brown MC Gordon S. The macrophage response to central and peripheral nerve injury. A possible role for macrophages in regeneration. J Exp Med. (1987) 165(4):1218–23. 10.1084/jem.165.4.1218 3559478 PMC2188570

[70] Brück W Huitinga I Dijkstra CD. Liposome-mediated monocyte depletion during Wallerian degeneration defines the role of hematogenous phagocytes in myelin removal. J Neurosci Res. (1996) 46(4):477–84. 10.1002/(SICI)1097-4547(19961115)46:4<477::AID-JNR9>3.0.CO;2-D 8950707

[71] Ydens E Amann L Asselbergh B Scott CL Martens L Sichien D Profiling peripheral nerve macrophages reveals two macrophage subsets with distinct localization, transcriptome and response to injury. Nat Neurosci. (2020) 23(5):676–89. 10.1038/s41593-020-0618-6 32284604 PMC7611025

[72] Lindborg JA Mack M Zigmond RE. Neutrophils are critical for myelin removal in a peripheral nerve injury model of Wallerian degeneration. J Neurosci. (2017) 37(43):10258–77. 10.1523/JNEUROSCI.2085-17.2017 28912156 PMC5656991

[73] Sollberger G Tilley DO Zychlinsky A. Neutrophil extracellular traps: the biology of chromatin externalization. Dev Cell. (2018) 44(5):542–53. 10.1016/j.devcel.2018.01.019 29533770

[74] Brinkmann V Reichard U Goosmann C Fauler B Uhlemann Y Weiss DS Neutrophil extracellular traps kill bacteria. Science. (2004) 303(5663):1532–5. 10.1126/science.1092385 15001782

[75] Vaibhav K Braun M Alverson K Khodadadi H Kutiyanawalla A Ward A Neutrophil extracellular traps exacerbate neurological deficits after traumatic brain injury. Sci Adv. (2020) 6(22):eaax8847. 10.1126/sciadv.aax8847 32523980 PMC7259928

[76] Yamamoto Y Kadoya K Terkawi MA Endo T Konno K Watanabe M Neutrophils delay repair process in Wallerian degeneration by releasing NETs outside the parenchyma. Life Sci Alliance. (2022) 5(10):e202201399. 10.26508/lsa.202201399 35961782 PMC9375156

[77] Denorme F Portier I Rustad JL Cody MJ de Araujo CV Hoki C Neutrophil extracellular traps regulate ischemic stroke brain injury. J Clin Invest. (2022) 132(10):e154225. 10.1172/JCI154225 35358095 PMC9106355

[78] Balog BM Niemi JP Disabato T Hashim F Zigmond RE. CXCR2 mediated trafficking of neutrophils and neutrophil extracellular traps are required for myelin clearance after a peripheral nerve injury. Exp Neurol. (2024) 382:114985. 10.1016/j.expneurol.2024.114985 39368532 PMC11526632

[79] Kanashiro A Hiroki CH da Fonseca DM Birbrair A Ferreira RG Bassi GS The role of neutrophils in neuro-immune modulation. Pharmacol Res. (2020) 151:104580. 10.1016/j.phrs.2019.104580 31786317 PMC7023896

[80] Kalinski AL Yoon C Huffman LD Duncker PC Kohen R Passino R Analysis of the immune response to sciatic nerve injury identifies efferocytosis as a key mechanism of nerve debridement. Elife. (2020) 9:e60223. 10.7554/eLife.60223 33263277 PMC7735761

[81] Van Hove H De Feo D Greter M Becher B. Central nervous system macrophages in health and disease. Annu Rev Immunol. (2025) 43(1):589–613. 10.1146/annurev-immunol-082423-041334 40036702

[82] Frosch M Prinz M. Niche-specific therapeutic targeting of myeloid cells in the central nervous system. Immunity. (2025) 58(5):1101–19. 10.1016/j.immuni.2025.03.016 40324377

[83] Amann L Prinz M. The origin, fate and function of macrophages in the peripheral nervous system-an update. Int Immunol. (2020) 32(11):709–17. 10.1093/intimm/dxaa030 32322888

[84] Dalmau Gasull A Glavan M Samawar SKR Kapupara K Kelk J Rubio M The niche matters: origin, function and fate of CNS-associated macrophages during health and disease. Acta Neuropathol. (2024) 147(1):37. 10.1007/s00401-023-02676-9 38347231 PMC10861620

[85] Stoll G Griffin JW Li CY Trapp BD. Wallerian degeneration in the peripheral nervous system: participation of both Schwann cells and macrophages in myelin degradation. J Neurocytol. (1989) 18(5):671–83. 10.1007/BF01187086 2614485

[86] Beuche W Friede RL. Myelin phagocytosis in Wallerian degeneration of peripheral nerves depends on silica-sensitive, bg/bg-negative and Fc-positive monocytes. Brain Res. (1986) 378(1):97–106. 10.1016/0006-8993(86)90289-1 3017506

[87] Wang PL Yim AKY Kim KW Avey D Czepielewski RS Colonna M Peripheral nerve resident macrophages share tissue-specific programming and features of activated microglia. Nat Commun. (2020) 11(1):2552. 10.1038/s41467-020-16355-w 32439942 PMC7242366

[88] Zhao XF Huffman LD Hafner H Athaiya M Finneran MC Kalinski AL The injured sciatic nerve atlas (iSNAT), insights into the cellular and molecular basis of neural tissue degeneration and regeneration. Elife. (2022) 11:e80881. 10.7554/eLife.80881 36515985 PMC9829412

[89] Chakarov S Lim HY Tan L Lim SY See P Lum J Two distinct interstitial macrophage populations coexist across tissues in specific subtissular niches. Science. (2019) 363(6432):1190. 10.1126/science.aau0964 30872492

[90] Masuda T Amann L Monaco G Sankowski R Staszewski O Krueger M Specification of CNS macrophage subsets occurs postnatally in defined niches. Nature. (2022) 604(7907):740–8. 10.1038/s41586-022-04596-2 35444273

[91] Beuche W Friede RL. The role of non-resident cells in Wallerian degeneration. J Neurocytol. (1984) 13(5):767–96. 10.1007/BF01148493 6512566

[92] Monaco S Gehrmann J Raivich G Kreutzberg GW. MHC-positive, ramified macrophages in the normal and injured rat peripheral nervous system. J Neurocytol. (1992) 21(9):623–34. 10.1007/BF01191724 1403008

[93] Lunn ER Perry VH Brown MC Rosen H Gordon S. Absence of Wallerian degeneration does not hinder regeneration in peripheral nerve. Eur J Neurosci. (1989) 1(1):27–33. 10.1111/j.1460-9568.1989.tb00771.x 12106171

[94] Chen HR Sun YY Chen CW Kuo YM Kuan IS Tiger Li ZR Fate mapping via CCR2-CreER mice reveals monocyte-to-microglia transition in development and neonatal stroke. Sci Adv. (2020) 6(35):eabb2119. 10.1126/sciadv.abb2119 32923636 PMC7449686

[95] Murray PJ Allen JE Biswas SK Fisher EA Gilroy DW Goerdt S Macrophage activation and polarization: nomenclature and experimental guidelines. Immunity. (2014) 41(1):14–20. 10.1016/j.immuni.2014.06.008 25035950 PMC4123412

[96] Ydens E Cauwels A Asselbergh B Goethals S Peeraer L Lornet G Acute injury in the peripheral nervous system triggers an alternative macrophage response. J Neuroinflammation. (2012) 9:176. 10.1186/1742-2094-9-176 22818207 PMC3419084

[97] Tomlinson JE Žygelytė E Grenier JK Edwards MG Cheetham J. Temporal changes in macrophage phenotype after peripheral nerve injury. J Neuroinflammation. (2018) 15(1):185. 10.1186/s12974-018-1219-0 29907154 PMC6003127

[98] Paolicelli RC Sierra A Stevens B Tremblay ME Aguzzi A Ajami B Microglia states and nomenclature: a field at its crossroads. Neuron. (2022) 110(21):3458–83. 10.1016/j.neuron.2022.10.020 36327895 PMC9999291

[99] Yim AKY Wang PL Bermingham JR Jr Hackett A Strickland A Miller TM Disentangling glial diversity in peripheral nerves at single-nuclei resolution. Nat Neurosci. (2022) 25(2):238–51. 10.1038/s41593-021-01005-1 35115729 PMC9060899

[100] Siebert H Sachse A Kuziel WA Maeda N Brück W. The chemokine receptor CCR2 is involved in macrophage recruitment to the injured peripheral nervous system. J Neuroimmunol. (2000) 110(1–2):177–85. 10.1016/S0165-5728(00)00343-X 11024548

[101] Perry VH Tsao JW Fearn S Brown MC. Radiation-induced reductions in macrophage recruitment have only slight effects on myelin degeneration in sectioned peripheral nerves of mice. Eur J Neurosci. (1995) 7(2):271–80. 10.1111/j.1460-9568.1995.tb01063.x 7538855

[102] Lindholm D Heumann R Meyer M Thoenen H. Interleukin-1 regulates synthesis of nerve growth factor in non-neuronal cells of rat sciatic nerve. Nature. (1987) 330(6149):658–9. 10.1038/330658a0 3317065

[103] Heumann R Korsching S Bandtlow C Thoenen H. Changes of nerve growth factor synthesis in nonneuronal cells in response to sciatic nerve transection. J Cell Biol. (1987) 104(6):1623–31. 10.1083/jcb.104.6.1623 3034917 PMC2114490

[104] Yeoh S Warner WS Merchant SS Hsu EW Agoston DV Mahan MA. Incorporating blood flow in nerve injury and regeneration assessment. Front Surg. (2022) 9:862478. 10.3389/fsurg.2022.862478 35529911 PMC9069240

[105] Gray M Palispis W Popovich PG van Rooijen N Gupta R. Macrophage depletion alters the blood-nerve barrier without affecting Schwann cell function after neural injury. J Neurosci Res. (2007) 85(4):766–77. 10.1002/jnr.21166 17266098

[106] Dai Z Liu WC Chen XY Wang X Li JL Zhang X. Gasdermin D-mediated pyroptosis: mechanisms, diseases, and inhibitors. Front Immunol. (2023) 14:1178662. 10.3389/fimmu.2023.1178662 37275856 PMC10232970

[107] Tao Y Wang F Xu Z Lu X Yang Y Wu J Gasdermin D in peripheral nerves: the pyroptotic microenvironment inhibits nerve regeneration. Cell Death Discov. (2021) 7(1):144. 10.1038/s41420-021-00529-6 34127647 PMC8203780

[108] Sugimoto MA Vago JP Perretti M Teixeira MM. Mediators of the resolution of the inflammatory response. Trends Immunol. (2019) 40(3):212–27. 10.1016/j.it.2019.01.007 30772190

[109] Pandey VK Acharya TK Willcox KF Dembla S Ramanujan A Grieco AR Peripheral nerve injury reduces macrophage efferocytosis to facilitate neuropathic pain. Proc Natl Acad Sci U S A. (2026) 123(1):e2511401122. 10.1073/pnas.2511401122 41481465 PMC12773720

[110] Kuhlmann T Bitsch A Stadelmann C Siebert H Brück W. Macrophages are eliminated from the injured peripheral nerve via local apoptosis and circulation to regional lymph nodes and the spleen. J Neurosci. (2001) 21(10):3401–8. 10.1523/JNEUROSCI.21-10-03401.2001 11331370 PMC6762479

[111] Mueller M Leonhard C Wacker K Ringelstein EB Okabe M Hickey WF Macrophage response to peripheral nerve injury: the quantitative contribution of resident and hematogenous macrophages. Lab Invest. (2003) 83(2):175–85. 10.1097/01.LAB.0000056993.28149.BF 12594233

[112] Davies AJ Kim HW Gonzalez-Cano R Choi J Back SK Roh SE Natural killer cells degenerate intact sensory afferents following nerve injury. Cell. (2019) 176(4):716–28.e18. 10.1016/j.cell.2018.12.022 30712871 PMC6418410

[113] Reel JM Abbadi J Cox MA. T cells at the interface of neuroimmune communication. J Allergy Clin Immunol. (2024) 153(4):894–903. 10.1016/j.jaci.2023.10.026 37952833 PMC10999355

[114] Cohen JA Edwards TN Liu AW Hirai T Jones MR Wu J Cutaneous TRPV1+ neurons trigger protective innate type 17 anticipatory immunity. Cell. (2019) 178(4):919–32.e14. 10.1016/j.cell.2019.06.022 31353219 PMC6788801

[115] Dhabhar FS Viswanathan K. Short-term stress experienced at time of immunization induces a long-lasting increase in immunologic memory. Am J Physiol Regul Integr Comp Physiol. (2005) 289(3):R738–44. 10.1152/ajpregu.00145.2005 15890793

[116] Rosas-Ballina M Olofsson PS Ochani M Valdés-Ferrer SI Levine YA Reardon C Acetylcholine-synthesizing T cells relay neural signals in a vagus nerve circuit. Science. (2011) 334(6052):98–101. 10.1126/science.1209985 21921156 PMC4548937

[117] Nordman JC Muldoon P Clark S Damaj MI Kabbani N. The α4 nicotinic receptor promotes CD4^+^ T-cell proliferation and a helper T-cell immune response. Mol Pharmacol. (2014) 85(1):50–61. 10.1124/mol.113.088484 24107512 PMC3868899

[118] Cao L DeLeo JA. CNS-infiltrating CD4^+^ T lymphocytes contribute to murine spinal nerve transection-induced neuropathic pain. Eur J Immunol. (2008) 38(2):448–58. 10.1002/eji.200737485 18196515 PMC2963094

[119] Raoof R Willemen HLDM Eijkelkamp N. Divergent roles of immune cells and their mediators in pain. Rheumatology. (2018) 57(3):429–40. 10.1093/rheumatology/kex308 28968842 PMC5850827

[120] Moalem G Xu K Yu L. T lymphocytes play a role in neuropathic pain following peripheral nerve injury in rats. Neuroscience. (2004) 129(3):767–77. 10.1016/j.neuroscience.2004.08.035 15541898

[121] Austin PJ Kim CF Perera CJ Moalem-Taylor G. Regulatory T cells attenuate neuropathic pain following peripheral nerve injury and experimental autoimmune neuritis. Pain. (2012) 153(9):1916–31. 10.1016/j.pain.2012.06.005 22789131

[122] Klein CJ Lennon VA Aston PA McKeon A Pittock SJ. Chronic pain as a manifestation of potassium channel-complex autoimmunity. Neurology. (2012) 79(11):1136–44. 10.1212/WNL.0b013e3182698cab 22895588 PMC3525306

[123] Dawes JM Weir GA Middleton SJ Patel R Chisholm KI Pettingill P Immune or genetic-mediated disruption of CASPR2 causes pain hypersensitivity due to enhanced primary afferent excitability. Neuron. (2018) 97(4):806–22.e10. 10.1016/j.neuron.2018.01.033 29429934 PMC6011627

[124] Lacagnina MJ Willcox KF Boukelmoune N Bavencoffe A Sankaranarayanan I Barratt DT B cells drive neuropathic pain-related behaviors in mice through IgG-Fc gamma receptor signaling. Sci Transl Med. (2024) 16(766):eadj1277. 10.1126/scitranslmed.adj1277 39321269 PMC11479571

[125] Fiore NT Willcox KF Grieco AR Dayani D Zuberi YA Heijnen CJ Autoreactive immunoglobulin G levels and Fc receptor gamma subunit upregulation drive mechanical allodynia after nerve constriction or crush injury. Pain. (2025) 166(12):2804–17. 10.1097/j.pain.0000000000003734 40728528 PMC12313113

[126] Cashman CR Hoke A. Deficiency of adaptive immunity does not interfere with Wallerian degeneration. PLoS One. (2017) 12(5):e0177070. 10.1371/journal.pone.0177070 28475650 PMC5419593

[127] Zigmond RE Echevarria FD. Macrophage biology in the peripheral nervous system after injury. Prog Neurobiol. (2019) 173:102–21. 10.1016/j.pneurobio.2018.12.001 30579784 PMC6340791

[128] Wynn TA Vannella KM. Macrophages in tissue repair, regeneration, and fibrosis. Immunity. (2016) 44(3):450–62. 10.1016/j.immuni.2016.02.015 26982353 PMC4794754

[129] Fertala J Rivlin M Wang ML Beredjiklian PK Steplewski A Fertala A. Collagen-rich deposit formation in the sciatic nerve after injury and surgical repair: a study of collagen-producing cells in a rabbit model. Brain Behav. (2020) 10(10):e01802. 10.1002/brb3.1802 32924288 PMC7559634

[130] Chiu IM Heesters BA Ghasemlou N Von Hehn CA Zhao F Tran J Bacteria activate sensory neurons that modulate pain and inflammation. Nature. (2013) 501(7465):52–7. 10.1038/nature12479 23965627 PMC3773968

[131] Kim BS Artis D. The sensory neuroimmune frontier. Immunity. (2025) 58(5):1033–9. 10.1016/j.immuni.2025.03.018 40324378

[132] Pinho-Ribeiro FA Deng L Neel DV Erdogan O Basu H Yang D Bacteria hijack a meningeal neuroimmune axis to facilitate brain invasion. Nature. (2023) 615(7952):472–81. 10.1038/s41586-023-05753-x 36859544 PMC10593113

[133] Lu YZ Nayer B Singh SK Alshoubaki YK Yuan E Park AJ CGRP sensory neurons promote tissue healing via neutrophils and macrophages. Nature. (2024) 628(8008):604–11. 10.1038/s41586-024-07237-y 38538784 PMC11023938

[134] Zhang X Zhang Y Chen Y Ji Y Lyu Y Miao Z Unraveling the immune system’s role in peripheral nerve regeneration: a pathway to enhanced healing. Front Immunol. (2025) 16:1540199. 10.3389/fimmu.2025.1540199 40061948 PMC11885135

[135] Mokarram N Merchant A Mukhatyar V Patel G Bellamkonda RV. Effect of modulating macrophage phenotype on peripheral nerve repair. Biomaterials. (2012) 33(34):8793–801. 10.1016/j.biomaterials.2012.08.050 22979988 PMC3483037

[136] Wasman Smail S Ziyad Abdulqadir S Omar Khudhur Z Elia Ishaq S Faqiyazdin Ahmed A Ghayour MB IL-33 promotes sciatic nerve regeneration in mice by modulating macrophage polarization. Int Immunopharmacol. (2023) 123:110711. 10.1016/j.intimp.2023.110711 37531832

[137] Qu WR Zhu Z Liu J Song DB Tian H Chen BP Interaction between Schwann cells and other cells during repair of peripheral nerve injury. Neural Regen Res. (2021) 16(1):93–8. 10.4103/1673-5374.286956 32788452 PMC7818858

[138] Li Y Zhang Z Xu K Du S Gu X Cao R Minocycline alleviates peripheral nerve adhesion by promoting regulatory macrophage polarization via the TAK1 and its downstream pathway. Life Sci. (2021) 276:119422. 10.1016/j.lfs.2021.119422 33781833

[139] Kiguchi N Kobayashi Y Saika F Sakaguchi H Maeda T Kishioka S. Peripheral interleukin-4 ameliorates inflammatory macrophage-dependent neuropathic pain. Pain. (2015) 156(4):684–93. 10.1097/j.pain.0000000000000097 25630024

[140] Vieira Paladino F de Moraes Rodrigues J da Silva A Goldberg AC. The immunomodulatory potential of Wharton’s jelly mesenchymal stem/stromal cells. Stem Cells Int. (2019) 2019:3548917. 10.1155/2019/3548917 31281372 PMC6594275

[141] Cheng Z Bosco DB Sun L Chen X Xu Y Tai W Neural stem cell-conditioned medium suppresses inflammation and promotes spinal cord injury recovery. Cell Transplant. (2017) 26(3):469–82. 10.3727/096368916X693473 27737726 PMC5657700

[142] Chen J Ren S Duscher D Kang Y Liu Y Wang C Exosomes from human adipose-derived stem cells promote sciatic nerve regeneration via optimizing Schwann cell function. J Cell Physiol. (2019) 234(12):23097–110. 10.1002/jcp.28873 31124125

[143] Bordett R Danazumi KB Wijekoon S Garcia CJ Abdulmalik S Kumbar SG. Advancements in stimulation therapies for peripheral nerve regeneration. Biomed Mater. (2024) 19(5):052008. 10.1088/1748-605X/ad651d PMC11425301 39025114

[144] Franks NP Lieb WR. Volatile general anaesthetics activate a novel neuronal K+ current. Nature. (1988) 333(6174):662–4. 10.1038/333662a0 2453807

[145] Koppes AN Nordberg AL Paolillo GM Goodsell NM Darwish HA Zhang L Electrical stimulation of Schwann cells promotes sustained increases in neurite outgrowth. Tissue Eng Part A. (2014) 20(3–4):494–506. 10.1089/ten.tea.2013.0012 24063574 PMC3926181

[146] Xu C Kou Y Zhang P Han N Yin X Deng J Electrical stimulation promotes regeneration of defective peripheral nerves after delayed repair intervals lasting under one month. PLoS One. (2014) 9(9):e105045. 10.1371/journal.pone.0105045 25181499 PMC4152131

[147] Yang H Datta-Chaudhuri T George SJ Haider B Wong J Hepler TD High-frequency electrical stimulation attenuates neuronal release of inflammatory mediators and ameliorates neuropathic pain. Bioelectron Med. (2022) 8(1):16. 10.1186/s42234-022-00098-8 36195968 PMC9533511

[148] Chang CJ Hsu SH. The effects of low-intensity ultrasound on peripheral nerve regeneration in poly(DL-lactic acid-co-glycolic acid) conduits seeded with Schwann cells. Ultrasound Med Biol. (2004) 30(8):1079–84. 10.1016/j.ultrasmedbio.2004.06.005 15474752

[149] Ren C Chen X Du N Geng S Hu Y Liu X Low-intensity pulsed ultrasound promotes Schwann cell viability and proliferation via the GSK-3beta/beta-catenin signaling pathway. Int J Biol Sci. (2018) 14(5):497–507. 10.7150/ijbs.22409 29805301 PMC5968842

[150] Yazdani SO Golestaneh AF Shafiee A Hafizi M Omrani HA Soleimani M. Effects of low level laser therapy on proliferation and neurotrophic factor gene expression of human Schwann cells in vitro. J Photochem Photobiol B. (2012) 107:9–13. 10.1016/j.jphotobiol.2011.11.001 22178388

[151] Ishiguro M Ikeda K Tomita K. Effect of near-infrared light-emitting diodes on nerve regeneration. J Orthop Sci. (2010) 15(2):233–9. 10.1007/s00776-009-1438-4 20358337

[152] StÖlting MN Arnold AS Haralampieva D Handschin C Sulser T Eberli D. Magnetic stimulation supports muscle and nerve regeneration after trauma in mice. Muscle Nerve. (2016) 53(4):598–607. 10.1002/mus.24780 26202157 PMC5130145

[153] Liu L Liu Z Huang L Sun Z Ma T Zhu S Pulsed magnetic field promotes proliferation and neurotrophic genes expression in Schwann cells in vitro. Int J Clin Exp Pathol. (2015) 8(3):2343–53. 26045741 PMC4440050

[154] Li R Liu J Li L Luo G Yuan X Shen S Porcine decellularized nerve matrix hydrogel attenuates neuroinflammation after peripheral nerve injury by inhibiting the TLR4/MyD88/NF-κB axis. Neural Regen Res. (2026) 21(3):1222–35. 10.4103/NRR.NRR-D-24-00302 39589179 PMC12296486

[155] Prest TA Yeager E LoPresti ST Zygelyte E Martin MJ Dong L Nerve-specific, xenogeneic extracellular matrix hydrogel promotes recovery following peripheral nerve injury. J Biomed Mater Res A. (2018) 106(2):450–9. 10.1002/jbm.a.36235 28891122 PMC5745279

[156] Bernard M McOnie R Tomlinson JE Blum E Prest TA Sledziona M Peripheral nerve matrix hydrogel promotes recovery after nerve transection and repair. Plast Reconstr Surg. (2023) 152(3):458e–67e. 10.1097/PRS.0000000000010261 36946873 PMC10461719

[157] Gao Y Wang Y Zhang J Zhang M Dai C Zhang Y Advancing neural regeneration via adaptable hydrogels: enriched with Mg^2+^ and silk fibroin to facilitate endogenous cell infiltration and macrophage polarization. Bioact Mater. (2024) 33:100–13. 10.1016/j.bioactmat.2023.10.026 38024231 PMC10658209

[158] Xu C Wu P Yang K Mu C Li B Li X Multifunctional biodegradable conductive hydrogel regulating microenvironment for stem cell therapy enhances the nerve tissue repair. Small. (2024) 20(23):e2309793. 10.1002/smll.202309793 38148305

[159] Jain N Moeller J Vogel V. Mechanobiology of macrophages: how physical factors coregulate macrophage plasticity and phagocytosis. Annu Rev Biomed Eng. (2019) 21:267–97. 10.1146/annurev-bioeng-062117-121224 31167103

[160] McWhorter FY Wang T Nguyen P Chung T Liu WF. Modulation of macrophage phenotype by cell shape. Proc Natl Acad Sci U S A. (2013) 110(43):17253–8. 10.1073/pnas.1308887110 24101477 PMC3808615

[161] Dong X Liu S Yang Y Gao S Li W Cao J Aligned microfiber-induced macrophage polarization to guide Schwann-cell-enabled peripheral nerve regeneration. Biomaterials. (2021) 272:120767. 10.1016/j.biomaterials.2021.120767 33813259

[162] Jia Y Yang W Zhang K Qiu S Xu J Wang C Nanofiber arrangement regulates peripheral nerve regeneration through differential modulation of macrophage phenotypes. Acta Biomater. (2019) 83:291–301. 10.1016/j.actbio.2018.10.040 30541701

[163] Sun Y Zhang Y Guo Y He D Xu W Fang W Electrical aligned polyurethane nerve guidance conduit modulates macrophage polarization and facilitates immunoregulatory peripheral nerve regeneration. J Nanobiotechnol. (2024) 22(1):244. 10.1186/s12951-024-02507-3 PMC11089704 38735969

[164] Wang JY Yuan Y Zhang SY Lu SY Han GJ Bian MX Remodeling of the intra-conduit inflammatory microenvironment to improve peripheral nerve regeneration with a neuromechanical matching protein-based conduit. Adv Sci. (2024) 11(17):e2302988. 10.4028/b-wjQRP6 PMC11077661 38430538

[165] Deumens R Bozkurt A Meek MF Marcus MAE Joosten EAJ Weis J Repairing injured peripheral nerves: bridging the gap. Prog Neurobiol. (2010) 92(3):245–76. 10.1016/j.pneurobio.2010.10.002 20950667

[166] Höke A. Mechanisms of disease: what factors limit the success of peripheral nerve regeneration in humans? Nat Clin Pract Neurol. (2006) 2(8):448–54. 10.1038/ncpneuro0262 16932603

[167] Gordon T. Peripheral nerve regeneration and muscle reinnervation. Int J Mol Sci. (2020) 21(22):8652. 10.3390/ijms21228652 33212795 PMC7697710

[168] Stenberg L Dahlin LB. Gender differences in nerve regeneration after sciatic nerve injury and repair in healthy and in type 2 diabetic Goto-Kakizaki rats. BMC Neurosci. (2014) 15:107. 10.1186/1471-2202-15-107 25216784 PMC4169809

[169] Mogil JS. Sex differences in pain and pain inhibition: multiple explanations of a controversial phenomenon. Nat Rev Neurosci. (2012) 13(12):859–66. 10.1038/nrn3360 23165262

[170] Gordon T Tyreman N Raji MA. The basis for diminished functional recovery after delayed peripheral nerve repair. J Neurosci. (2011) 31(14):5325–34. 10.1523/JNEUROSCI.6156-10.2011 21471367 PMC6622714

[171] Kornfeld T Vogt PM Radtke C. Nerve grafting for peripheral nerve injuries with extended defect sizes. Wien Med Wochenschr. (2019) 169(9–10):240–51. 10.1007/s10354-018-0675-6 30547373 PMC6538587

[172] Grinsell D Keating CP. Peripheral nerve reconstruction after injury: a review of clinical and experimental therapies. Biomed Res Int. (2014) 2014:698256. 10.1155/2014/698256 25276813 PMC4167952

[173] Carnicer-Lombarte A Chen ST Malliaras GG Barone DG. Foreign body reaction to implanted biomaterials and its impact in nerve neuroprosthetics. Front Bioeng Biotechnol. (2021) 9:622524. 10.3389/fbioe.2021.622524 33937212 PMC8081831

[174] di Summa PG Kingham PJ Raffoul W Wiberg M Terenghi G Kalbermatten DF. Adipose-derived stem cells enhance peripheral nerve regeneration. J Plast Reconstr Aesthet Surg. (2010) 63(9):1544–52. 10.1016/j.bjps.2009.09.012 19828391

[175] Al-Majed AA Brushart TM Gordon T. Electrical stimulation accelerates and increases expression of BDNF and trkB mRNA in regenerating rat femoral motoneurons. Eur J Neurosci. (2000) 12(12):4381–90. 10.1046/j.1460-9568.2000.01341.x 11122348

[176] Costello MC Errante EL Smartz T Ray WZ Levi AD Burks SS. Clinical applications of electrical stimulation for peripheral nerve injury: a systematic review. Front Neurosci. (2023) 17:1162851. 10.3389/fnins.2023.1162851 37600003 PMC10435250

[177] Hoben GM Ee X Schellhardt L Yan Y Hunter DA Moore AM Increasing nerve autograft length increases senescence and reduces regeneration. Plast Reconstr Surg. (2018) 142(4):952–61. 10.1097/PRS.0000000000004759 29994844 PMC6156921

[178] Isaacs J Mallu S Yan W Little B. Consequences of oversizing: nerve-to-nerve tube diameter mismatch. J Bone Joint Surg Am. (2014) 96(17):1461–7. 10.2106/JBJS.M.01420 25187585

